# 2,2,3,3,4,4,4-Heptafluorobutyl Acetate—Chemical Equilibrium and Kinetics of the Esterification Reaction of 2,2,3,3,4,4,4-Heptafluorobutan-1-ol and Acetic Acid in the Presence of an Acidic Catalyst

**DOI:** 10.3390/molecules30081744

**Published:** 2025-04-13

**Authors:** Andrei V. Polkovnichenko, Evgenia I. Kovaleva, Viktor I. Privalov, Nikita A. Selivanov, Sergey Ya. Kvashnin, Egor V. Lupachev

**Affiliations:** Kurnakov Institute of General and Inorganic Chemistry RAS, Leninskii Prospekt, 31, 119991 Moscow, Russia; evg.kowalewa2012@yandex.ru (E.I.K.); privalov@rambler.ru (V.I.P.); goovee@yandex.ru (N.A.S.); kvashnins@bk.ru (S.Y.K.); egorlu91@gmail.com (E.V.L.)

**Keywords:** esterification, 2,2,3,3,4,4,4-heptafluorobutan-1-ol, 2,2,3,3,4,4,4-heptafluorobutyl acetate, acetic acid, chemical kinetic, chemical equilibrium, Arrhenius equation parameters

## Abstract

The kinetics and chemical equilibrium of the esterification reaction of acetic acid (AAc) and 2,2,3,3,4,4,4-heptafluorobutan-1-ol (HFBol) (using sulfuric acid as a catalyst) are determined experimentally. The study presents the dependences of $K_{eq}$ on the initial molar ratio of reagents, catalyst concentration and temperature. It is shown that all of the above parameters significantly affect the chemical equilibrium of the system. According to the Van’t Hoff equation, the standard enthalpy and standard entropy are calculated from the experimental data. The esterification process of AAc and HFBol is characterized by the negative heat effect (${∆}_{r}H$ > 0). The homogeneous and heterogeneous regions of the chemical equilibrium composition at different settings are given. In the homogeneous region of the chemical equilibrium composition, it is found that the rate constant and half-reaction time do not depend on the initial molar ratio of the reagents. The dependencies of the rate constant on the temperature are obtained, and the parameters of the Arrhenius equation are estimated from the experiments.

## 1. Introduction

Organofluorine compounds which have carbon-fluorine bonds show unique features such as high thermal and chemical stability, high surface activity, no light-absorbing ability, high pharmacological effect, etc. [1,2] and are applied in various industries, generating significant interest among researchers. The key aspect of the organofluorine compound industry is the search and design of new synthesis methods and production technologies, particularly owing to the high cost of such products. Meanwhile, organofluorine substances, including organofluoric esters, still remain much less studied than their hydrocarbon analogues.

This study is a part of the scientific project and specifically targets the chemistry of 2,2,3,3,4,4,4-heptafluorobutyl acetate (HFBAc) and the specifics behind its production technology. The product itself is used in the manufacture of non-aqueous electrolytes, ethyllithium sulfate, charge retention mediums, ultraviolet light-absorbing oligomers, and more [3,4,5,6,7].

One of the key methods for the ester production nowadays is the reactive distillation process, which is based on the equilibrium reaction. By integrating the chemical reaction and separation processes into a single apparatus, the reactive distillation significantly simplified the process and allows to overcome thermodynamic limitations, achieving high conversion rates, selectivity, and product yields [8,9,10]. In the present study, the esterification of 2,2,3,3,4,4,4-heptafluorobutan-1-ol (HFBol) and acetic acid (AAc) under acidic conditions is considered as the basis for reactive distillation:

(1) $$\begin{array}{c} C_{4}F_{7}H_{2}OH+CH_{3}COOH↔{CH}_{3}COOC_{4}F_{7}H_{2}+H_{2}O \end{array}$$

To design the method of the HFBAc production, the data on the properties and the specifics of the reactive system underlying the reactive distillation are required. Such data are not available in the scientific literature for the system under research.

The aim of the present study is to investigate the chemical equilibrium and kinetics of the esterification reaction of AAc and HFBol under acidic conditions.

## 2. Experimental Results and Discussion

### 2.1. Chemical Equilibrium

#### 2.1.1. Dependence of the Equilibrium Constant on the Initial Molar Ratio of the Reagents

**Figure 1:**
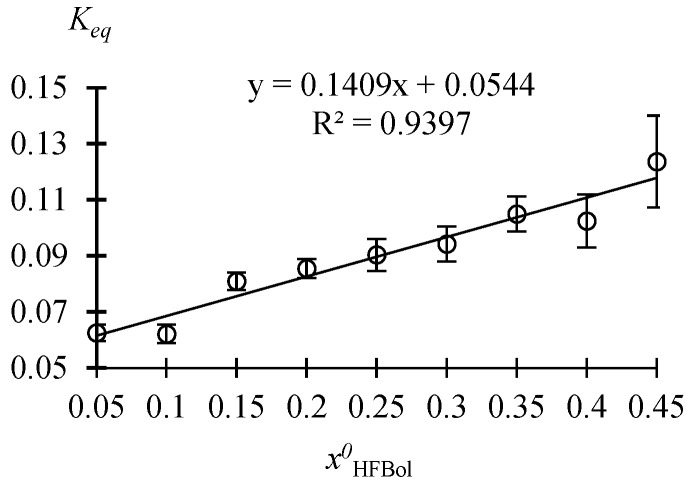
Dependence of the equilibrium constant $K_{eq}$ on the initial molar ratio of the reagents at atmospheric pressure, *T* = 50 °C and $x_{H_{2}SO_{4}}=0.01\pm 0.0007$ mole fr. according to the Table 1 data.

The dependence of the composition of the reaction mixture at different initial molar ratios of the reagents on the time of thermostating at atmospheric pressure, $x_{H_{2}SO_{4}}=0.01$ mole fr. and *T* = 50 °C, is given in the Supplementary Materials File (Table S1). Data are processed and summarized in Table 1 and Figure 1.

From the data of Table 1, it follows that at $x_{H_{2}SO_{4}}=0.01$ mole fr., T = 50 °C and $x_{HFBol}^{0}$ ≤ 0.35 mole fr., the reaction system HFBol–AAc–HFBAc–water belongs to the homogeneous region of chemically equilibrium compositions; and at $x_{HFBol}^{0}$ ≥ 0.40 mole fr., it belongs to the heterogeneous region of chemically equilibrium compositions. The presence of the heterogeneous region is caused by the limited mutual solubility of water and HFBol and HFBAc.

From the data of Figure 1, it can be seen that the $K_{eq}$ value of the esterification reaction of AAc and HFBol depends significantly on the initial molar ratio of the reactants. At atmospheric pressure, temperature 50 °C and $x_{H_{2}SO_{4}}=0.01$ mole fr., the $K_{eq}$ values increase linearly as $x_{HFBol}^{0}$ increases and can be described by Equation (2):

(2) $$\begin{array}{c} K_{eq}=0.1409x_{HFBol}^{0}+0.0544 \end{array}$$

where $x_{HFBol}^{0}$ is the initial mole fraction of HFBol.

Similar dependences of the equilibrium constants calculated from the concentrations on the initial ratio of reactants were obtained for the esterification reactions of acetic acid with ethanol, propionic acid with ethanol and acetic acid with butanol-1 [11,12].

#### 2.1.2. Dependence of the Equilibrium Constant on the Catalyst Concentration

**Figure 2:**
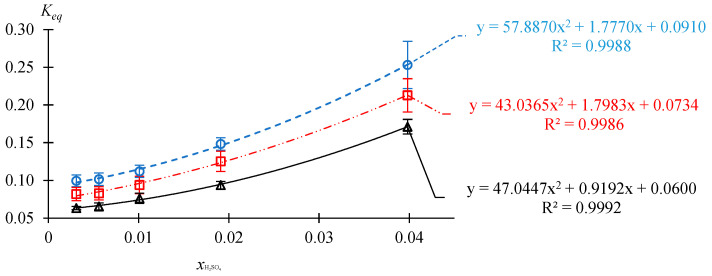
Dependence of the equilibrium constant $K_{eq}$ on the catalyst concentration at atmospheric pressure, initial molar ratio AAc/HFBol = 65/35 and different temperatures according to the Table 2 data. ○—70 °C; ☐—50 °C; ∆—30 °C.

The dependence of the composition of the reaction mixture at different catalyst concentrations on the time of thermostating at atmospheric pressure, initial molar ratio AAc/HFBol = 65/35 and *T* = 30, 50 and 70 °C is given in the Supplementary Materials File (Table S2). Data are processed and summarized in Table 2 and Figure 2.

From the data of Table 2, it follows that at initial molar ratio AAc/HFBol = 65/35, temperature range from 30 to 70 °C and $x_{H_{2}SO_{4}}\leq 0.0101$ mole fr., the reaction system HFBol–AAc–HFBAc–water belongs to the homogeneous region of chemically equilibrium compositions; and at $x_{H_{2}SO_{4}}$ ≥ 0.0191 mole fr., it belongs to the heterogeneous region of chemically equilibrium compositions.

From the data of Figure 2, it can be seen that the $K_{eq}$ value of the esterification reaction of AAc and HFBol at initial molar ratio AAc/HFBol = 65/35 depends significantly on the catalyst concentration for all of the studied temperatures. The dependence can be described by Equation (3):

(3) $$\begin{array}{c} \begin{array}{c} T=70\;{}^{\circ}C;\;K_{eq}=57.8870{\left(x_{H_{2}SO_{4}}\right)}^{2}+1.7770x_{H_{2}SO_{4}}+0.0910\\ T=50\;{}^{\circ}C;\;K_{eq}=43.0365{\left(x_{H_{2}SO_{4}}\right)}^{2}+1.7983x_{H_{2}SO_{4}}+0.0734\\ T=30\;{}^{\circ}C;\;K_{eq}=47.0447{\left(x_{H_{2}SO_{4}}\right)}^{2}+0.9192x_{H_{2}SO_{4}}+0.0600 \end{array} \end{array}$$

where $x_{H_{2}SO_{4}}$ is the catalyst mole fraction.

#### 2.1.3. Dependence of the Equilibrium Constant on Temperature

**Figure 3:**
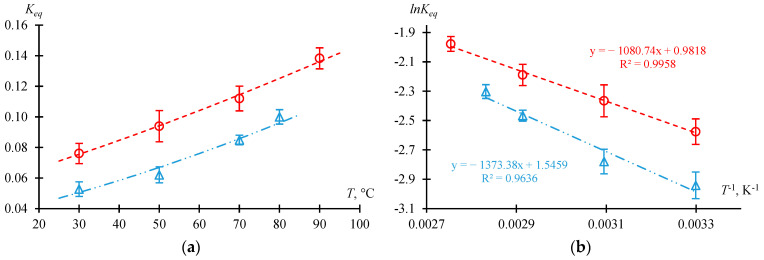
Dependence of the equilibrium constant $K_{eq}$ on temperature *T* at $x_{H_{2}SO_{4}}=0.01$ mole fr. and atmospheric pressure according to the Table 3 data. (**a**) Linear coordinates, (**b**) Arrhenius coordinates. ○—initial molar ratio AAc/HFBol = 65/35; ∆—initial molar ratio AAc/HFBol = 9/1; line—model.

**Figure 4:**
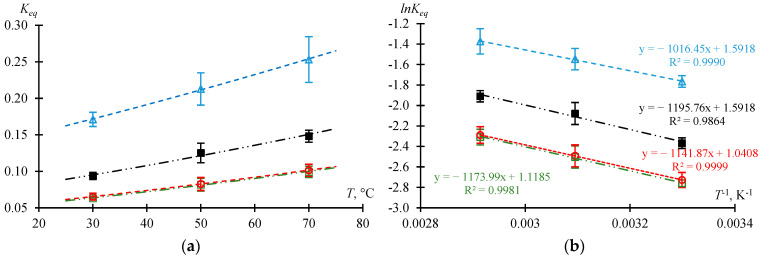
Dependence of the equilibrium constant $K_{eq}$ on the temperature *T* at initial molar ratio AAc/HFBol = 65/35 and atmospheric pressure according to the data in Table 2 and Table 3. (**a**) Linear coordinates, (**b**) Arrhenius coordinates. ☐—$x_{H_{2}SO_{4}}=0.0031$ mole fr.; ○—$x_{H_{2}SO_{4}}=0.0056$ mole fr.; ■—$x_{H_{2}SO_{4}}=0.0191$ mole fr.; ∆—$x_{H_{2}SO_{4}}=0.0398$ mole fr.; line—model.

The dependence of the composition of the reaction mixture at different temperatures on the time of thermostating is given in the Supplementary Materials File. For the initial molar ratio AAc/HFBol = 9/1 at atmospheric pressure and $x_{H_{2}SO_{4}}=0.01$ mole fr., data are summarized in Table S3; for the initial molar ratio AAc/HFBol = 65/35 and catalyst concentration $x_{H_{2}SO_{4}}$ = [0.031; 0.0398] mole fr., data are summarized in Tables S2 and S4. Experimental data were processed and are presented in Table 2 and Table 3 as well as Figure 3 and Figure 4.

Figure 3 and Figure 4 illustrate the dependence of $K_{eq}$ on temperature. The dots represent the experimental values, and the lines represent the obtained models. Figure 3 shows the dependence of $K_{eq}$ on the initial molar ratio of the reactants in a wide range of temperatures. The data are consistent with the results of the previous section: $K_{eq}$ increases as $x_{HFBol}^{0}$ increases. It is also to be noted that the dependence of $K_{eq}$ on the temperature at $x_{H_{2}SO_{4}}=0.01$ mole fr. for initial molar ratio AAc/HFBol = 9/1 and 65/35 is characterized by a constant difference $∆K_{eq}$ = 0.027 ± 0.003. From the data in Figure 3 and Figure 4, the Van’t Hoff equation (Equation (15)) parameters can be estimated: the slope is $\tan\alpha ={∆}_{r}H/R$, and the intercept is ${∆}_{r}S/R$. The calculated values of the standard enthalpy and standard entropy of the esterification reaction of AAc and HFBol are given in Table 4. Table 4 also provides equations that allow to calculate the value of $K_{eq}$ for given conditions in the temperature range from 30 to 90 °C.

According to the Table 4 data for the investigated range of conditions, the esterification process of AAc and HFBol is characterized by a negative heat effect (${∆}_{r}H$ > 0). In general, the heat effect of the esterification process depends on the individual component’s properties and on the unique system properties as a whole. So, the process can be either endothermic [13] or exothermic [14]. It should be considered that the total heat effect of the esterification process of AAc and HFBol, in addition to the heat effect of the reaction itself, includes the hydrophobic effects of HFBol [15] and HFBAc as well as hydration of sulfuric and acetic acids due to the water formation in the system.

**Figure 5:**
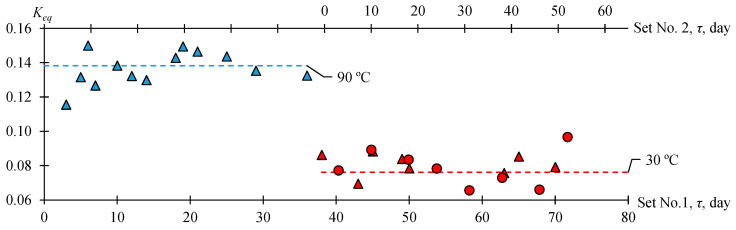
Dependence of the equilibrium constant $K_{eq}$ on temperature at initial molar ratio AAc/HFBol = 65/35, $x_{H_{2}SO_{4}}=0.01$ mole fr. and atmospheric pressure. ∆—set No. 1; ○—set No. 2; line—$K_{eq}^{av}$. Blue—90 °C; red—30 °C.

To confirm the obtained data, an additional experiment with two parallel sets was run (Figure 5). Sample 1 was thermostated at 90 °C for 36 days, after which the temperature was reduced to 30 °C and the sample continued to be thermostated for a further 34 days (70 days in total). Set 2 was thermostated at 30 °C for 52 days. From the data in Figure 5, it follows that $K_{eq}$ decreases as the temperature decreases or, vice versa, $K_{eq}$ increases as the temperature increases, and the period of temperature conditioning of samples is sufficient to reach chemical equilibrium.

### 2.2. Chemical Kinetic

**Figure A1:**
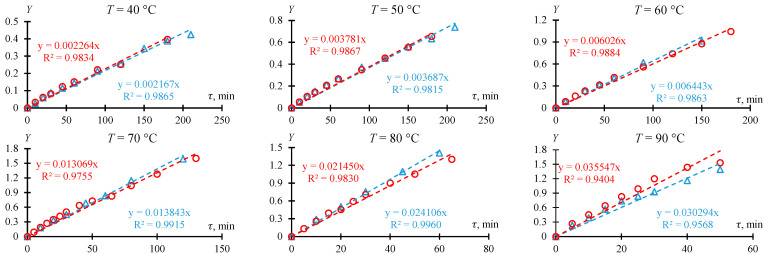
Dependence of the function Y on time *τ* (Equation (19)) at initial molar ratio AAc/HFBol = 9/1, $x_{H_{2}SO_{4}}=0.01$ mole fr. and atmospheric pressure (laboratory stirred reactor). Dots—experimental data according to Equation (19) calculated from the Table S5 data. ∆—set No. 1; ○—set No. 2. Used in Equation (19), and the $K_{eq}$ value is calculated by Equation (4).

**Figure A2:**
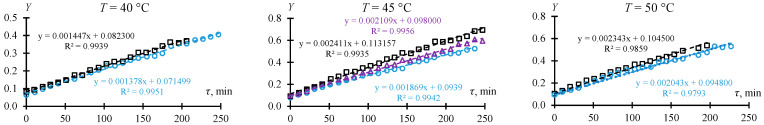

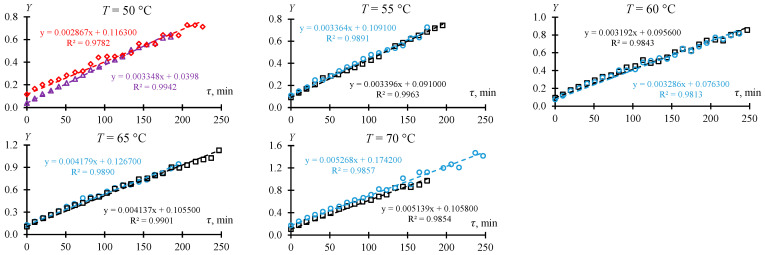
Dependence of the function Y on time *τ* (Equation (19)) at initial molar ratio AAc/HFBol = 9/1, $x_{H_{2}SO_{4}}=0.01$ mole fr. and atmospheric pressure (NMR apparatus). Dots—experimental data according to the Equation (19) calculated from the Table S6 data. ○—set 1; ☐—set 2; ∆—set 3; 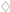
—set 4. Used in Equation (19), and the $K_{eq}$ value is calculated by Equation (4).

**Figure A3:**
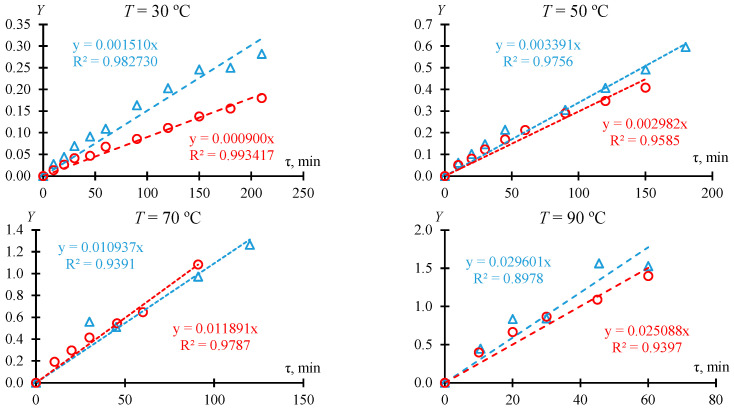
Dependence of the function Y on time *τ* (Equation (19)) at initial molar ratio AAc/HFBol = 65/35, $x_{H_{2}SO_{4}}=0.01$ mole fr. and atmospheric pressure (laboratory stirred reactor). Dots—experimental data according to the Equation (19) calculated from the Table S7 data. ∆—set No. 1; ○—set No. 2. Used in Equation (19), and the $K_{eq}$ value is calculated by Equation (5).

The kinetics of the AAc and HFBol esterification reaction was studied in the homogeneous region of chemically equilibrium compositions, namely for the initial molar ratio of the reactants AAc/HFBol = 9/1 and 65/35 at the catalyst concentration $x_{H_{2}SO_{4}}=0.01$ mole fr. and the temperature range from 30 to 90 °C. The experiments were carried out in the laboratory stirred reactor; the equipment and methodology are described above. For the initial molar ratio of the reactants AAc/HFBol = 9/1, data were additionally obtained using a methodology similar to that presented in [16]: the reaction is carried out in the NMR tube directly in the NMR apparatus (Bruker Avance II—300 MHz NMR spectrometer) with no stirring. Just before the sample was loaded into the NMR apparatus, the sulfuric acid was added to the NMR tube with a sample of known composition and turned over several times to mix the components. No deuterated solvent was used. After the tube was loaded into the spectrometer, the designed temperature was set. After the required temperature was reached at specified intervals, the NMR spectra were recorded. NMR spectra were recorded every 10 min; for each NMR spectrum, the relaxation delay is 0.4 s, the pre-scan delay is 8 µs, the dwell time is 1.2 µs, the acquisition time is 0.078 s, and the high power pulse is 4 µs. Corresponding comments have been added to the paper. The experimental dataset of the dependence of the composition of the reaction mixture at different temperatures on the time of thermostating is given in the Supplementary Materials File: at the initial molar ratio AAc/HFBol = 9/1 in the laboratory stirred reactor in Table S5; at the initial molar ratio AAc/HFBol = 9/1 in the NMR apparatus in Table S6; and at the initial molar ratio AAc/HFBol = 65/35 in the laboratory stirred reactor in Table S7. The data in Tables S5–S7 are processed by Equation (19), presented in Figure A1, Figure A2 and Figure A3 and summarized in Table 5, Table 6 and Table 7.

**Figure 6:**
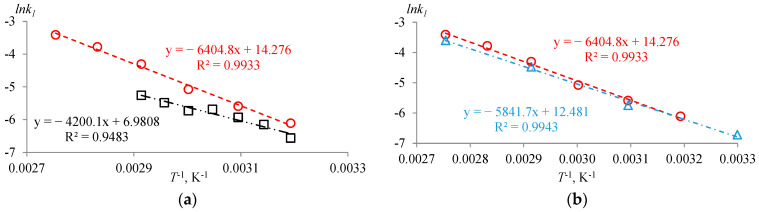
Dependence of the rate constant $k_{1}$ of the esterification reaction of AAc and HFBol on the temperature *T* in Arrhenius coordinates at $x_{H_{2}SO_{4}}=0.01$ mole fr. and atmospheric pressure. (**a**) Laboratory stirred reactor vs. NMR apparatus at initial molar ratio AAc/HFBol = 9/1; (**b**) at different initial molar ratio in the laboratory stirred reactor. ☐—initial molar ratio AAc/HFBol = 9/1 in NMR apparatus (Table 6); ○—initial molar ratio AAc/HFBol = 9/1 in laboratory stirred reactor (Table 5); ∆—initial molar ratio AAc/HFBol = 65/35 in laboratory stirred reactor (Table 7).

From the Figure A1, Figure A2 and Figure A3 data and experimental dataset from Tables S5–S7, it follows that the experimental results for the parallel sets, except that where the catalyst concentration differ considerably (Figure A3, 30 °C), are in rather good agreement. Table 5, Table 6 and Table 7 represent the dependence of the rate constant on the reaction time and Arrhenius coordinates, which are shown in Figure 6.

From Figure 6a, it is evident that stirring significantly affects the reaction rate of AAc and HFBol. At $x_{H_{2}SO_{4}}=0.01$ mole fr., the process in the experiments in the NMR apparatus is limited by diffusion. So, it can be concluded that the experimental approach, in which the reaction is carried out in the NMR tube directly in the NMR apparatus, is invalid for estimation of the reaction kinetics. Nevertheless, such data may be useful for the evaluation of diffusion processes in the system. From the Figure 6b data, it can be seen that the initial ratio of reagents in the investigated temperature range does not affect significantly the value of the rate constant of the esterification reaction of AAc and HFBol. It is interesting to note that despite the significant difference in the $K_{eq}$ values, the half-reaction time $\tau^{1/2}$ is independent of the initial molar ratio of the reactants within the experimental uncertainty (Table 8).

Based on the dependence of $k_{1}$ on temperature (Figure 6), the parameters of the Arrhenius equation (Equation (21)) are calculated and summarized in Table 9. For data obtained in the laboratory stirred reactor at initial molar ratios of AAc/HFBol = 9/1 and 65/35, the parameters were estimated both separately and as the merge dataset. To plot the mathematical model, the apparent parameters of Arrhenius equation for the reaction in the NMR apparatus are also provided.

**Figure A4:**
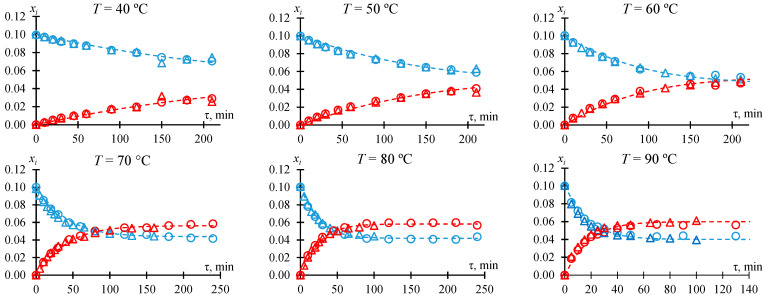
Dependence of the composition x_i_ of the reaction mixture on time *τ* at initial molar ratio AAc/HFBol = 9/1, $x_{H_{2}SO_{4}}=0.01$ mole fr. and atmospheric pressure (laboratory stirred reactor). Dots—experimental data according to Table S5: ○—set 1; ∆—set 2; red—HFBAc; blue—HFBol. Lines—model calculated by Equations (19) and (10).

**Figure A5:**
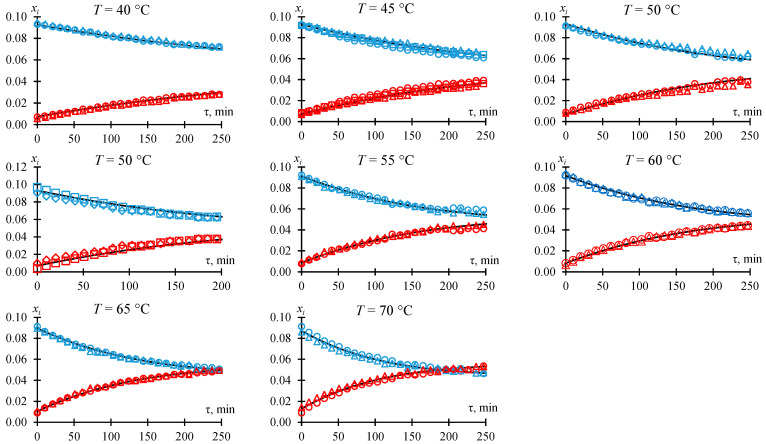
Dependence of the composition x_i_ of the reaction mixture on time *τ* at initial molar ratio AAc/HFBol = 9/1, $x_{H_{2}SO_{4}}=0.01$ mole fr. and atmospheric pressure (NMR apparatus). Dots—experimental data according to Table S6: ○—set 1; ∆—set 2; ☐—set 3; 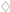
—set 4. red—HFBAc; blue—HFBol. Lines—model calculated by Equations (19) and (11).

**Figure A6:**
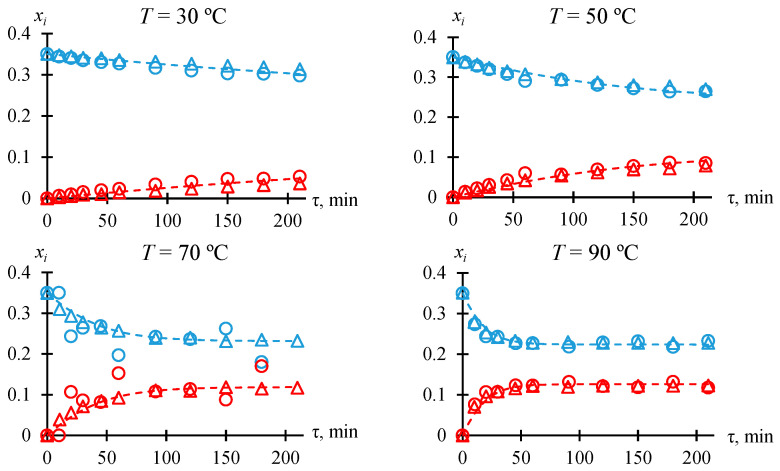
Dependence of the composition x_i_ of the reaction mixture on time *τ* at initial molar ratio AAc/HFBol = 65/35, $x_{H_{2}SO_{4}}=0.01$ mole fr. and atmospheric pressure (laboratory stirred reactor). Dots—experimental data according to Table S7: ○—set 1; ∆—set 2; red—HFBAc; blue—HFBol. Lines—model calculated by Equations (19) and (12).

From the data in Table 9, it follows that despite the low $E_{a}$ of the esterification reaction in the NMR apparatus, the pre-exponential factor, which characterizes the number of collisions, is several orders lower than those in the experiments obtained in the laboratory stirred reactor. The parameters of the Arrhenius equation at initial molar ratios of AAc/HFBol = 9/1 and 65/35 in the laboratory stirred reactor are close; the values given in Table 9 are comparable with the literature data for esterification reactions [17,18,19]. In general, the obtained mathematical model adequately describes the experimental data related to the dependence of the reaction mixture composition on the reaction time (Figure A4, Figure A5 and Figure A6).

## 3. Materials and Methods

HFBol was provided by P&M Invest (Moscow, Russia) with the initial purity of the compound about 0.60–0.90 mass fr. The substance was purified by distillation and heteroazeotropic distillation in the presence of various separating agents. The final purity of HFBol was determined by gas chromatography (an Agilent 6890 N equipped with a Restek RTX-1701 RK12054 capillary column; Agilent Technologies, Inc., Wilmington, DE, USA). AAc was used without further purification. As a catalyst in the work, sulfuric acid was used. Dimethyl sulfoxide d-6 (DMSO-d6) was used as a solvent for NMR analysis. The compounds used in this work, along with their final purity, are presented in Table 10.

**Figure 7:**
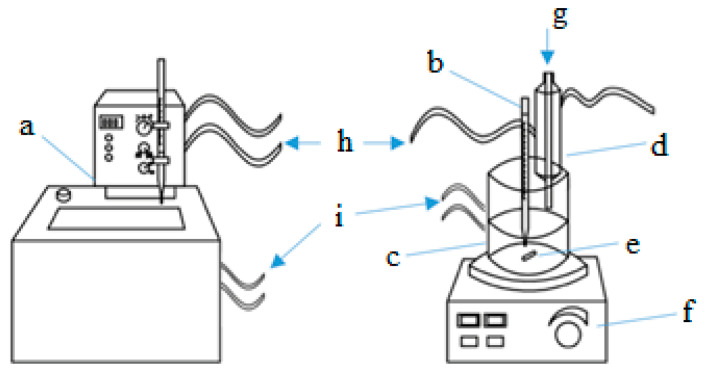
Equipment for researching the kinetics of a chemical reaction. a—thermostat, b—mercury thermometer, c—laboratory reactor with jacket, d—condenser, e—stirrer bar, f—magnetic stirrer, g—sample collection, h—cooling medium, i—heat-carrying medium.

The equilibrium constant ($K_{eq}$—Equation (14)) of the esterification reaction of AAc and HFBol was measured by the continuous thermostating of samples at atmospheric pressure with a known initial molar ratio of reagents and catalyst concentration. Here, the following should be pointed out to the readers. In the present study, the chemical equilibrium is described by the equilibrium constant calculated from the concentration. In the temperature range from 30 to 70 °C, the samples were thermostated in the thermo-cabinet TS-1/20 SPU from «MedComplect A.V.K.» (Moscow, Russia), while for temperatures over 70 °C, they were thermostated in a reactor with a stirrer (Figure 7). The samples were thermostated until the composition of the mixture was no longer changed and the fluctuations in the reaction mixture composition over time reached constant values within the experiment uncertainty. For a number of conditions, a series of several parallel experiments was performed to validate and to support the data. In the latter case, the equilibrium constant is calculated as the average one across the series parallel experiments ($K_{eq}^{av}$—Equation (15)).

(14) $$\begin{array}{c} K_{eq}=\frac{k_{1}}{k_{2}}=\frac{x_{HFBAc}x_{H_{2}O}}{x_{HFBol}x_{AAc}} \end{array}$$

where $x_{i}$ is the mole fraction of component *i*; $k_{1}$ and $k_{2}$ are the rate constants of the forward and reverse reaction in mole fr.^−1^·min^−1^, respectively.

(15) $$\begin{array}{c} K_{eq}^{av}=\frac{\sum_{i=1}^{n}K_{eq}^{i}}{n} \end{array}$$

where *n* is the number of parallel experiments.

The dependence of the $K_{eq}$ on temperature *T* is described by the Van’t Hoff equation (Equation (16)). The dependence $lnK_{eq}=f\left(\frac{1}{T}\right)$ indicates the standard enthalpy and standard entropy of the reaction.

(16) $$\begin{array}{c} lnK_{eq}=-\frac{\Delta_{r}H}{RT}+\frac{\Delta_{r}S}{R} \end{array}$$

where $\Delta_{r}H$ is standard enthalpy of the reaction, J·mol^−1^; $\Delta_{r}S$ is the standard entropy of the reaction, J·mol^−1^·K^−1^; *R* is the gas constant, 8.314 J·mol^−1^·K^−1^; and *T* is the temperature in K.

The kinetic experiment apparatus is a laboratory stirred reactor (Figure 7). All the experiments are performed at atmospheric pressure. Since the esterification of AAc and HFBol (Equation (1)) does not occur without a catalyst, the initial mixture of reagents of known ratio with a volume of 50 mL is loaded into the reactor and heated with constant stirring to working temperature. Once the temperature has been reached, sulfuric acid is introduced into the system; this moment is considered to be the starting point of the experiment *τ* = 0. Further, the reaction mixture is sampled at specified intervals. To validate and to support the data, each experiment was repeated at least twice.

The samples were quantitatively analyzed using NMR. The Bruker Avance II—300 MHz NMR spectrometer (Bruker Corp., Billerica, MA, USA) was employed to obtain ^1^H and ^19^F spectra of the samples at frequencies of 300.211 and 282.499 MHz, respectively, using an internal deuterium lock. We used a standard method to calculate the quantitative composition from NMR spectra. The NMR spectra of HFBol and HFBAc were published in our previous paper [20]. The ratio of HFBAc (CF_3_**CF_2_**CF_2_CH_2_OOCH_3_—shift is −121.57 ppm) to HFBol (CF_3_**CF_2_**CF_2_CH_2_OH—shift is −123.60 ppm) was determined using the ^19^F spectrum, while the ^1^H spectrum was used to determine the ratio of HFBAc (CF_3_CF_2_CF_2_CH_2_OO**CH_3_**—shift is 2.16 ppm) to AAc (**CH_3_**COOH—shift is 1.93 ppm). In calculations, the concentration of water was taken to be equal to the concentration of HFBAc.

A second-order reaction kinetic model is taken to describe the esterification reaction of AAc and HFBol. The reaction rate v in this case is defined as

(17) $$\begin{array}{c} v=\frac{dx}{d\tau }=k_{1}x_{HFBol}x_{AAc}-k_{2}x_{HFBAc}x_{H_{2}O} \end{array}$$

where *x_i_* is the mole fraction of component *i* at the time moment *τ*; *τ* is the reaction time in min.

Considering Equation (14), it follows that

(18) $$\begin{array}{c} k_{1}d\tau =\frac{dx}{x_{HFBol}x_{AAc}-\frac{1}{K_{eq}}x_{HFBAc}x_{H_{2}O}} \end{array}$$

The left part of Equation (18) is integrated between 0 and τ, and the right part is integrated between 0 and $x_{HFBAc}$. Considering that at any moment of time *τ*, the $x_{HFBAc}{=x}_{H_{2}O}$, $x_{HFBol}=x_{HFBol}^{0}-x_{HFBAc}$ and $x_{Ac}=x_{AAc}^{0}-x_{HFBAc}$, where $x_{i}^{0}$ is the mole fraction of component *i* at *τ* = 0, the following is obtained:

(19) $$\begin{array}{c} k_{1}\tau =\frac{1}{\sqrt{C}}ln\left[\frac{2x_{HFBol}^{0}x_{AAc}^{0}-x_{HFBAc}\left(x_{HFBol}^{0}+x_{Ac}^{0}-\sqrt{C}\right)}{2x_{HFBol}^{0}x_{AAc}^{0}-x_{HFBAc}\left(x_{HFBol}^{0}+x_{Ac}^{0}+\sqrt{C}\right)}\right]=Y \end{array}$$

where

(20) C=xHFBol0+xAAc02−4xHFBol0xAAc01−1Keq

For the second-order reaction, the dependence Y=f(τ) from Equation (19) is linear, and the slope of the line is equal to the rate constant of the forward reaction $k_{1}$. The dependence of the rate constant $k_{1}$ on temperature *T* is given by the Arrhenius equation:

(21) $$\begin{array}{c} lnk=\frac{-E_{a}}{RT}+lnA \end{array}$$

where $E_{a}$ is the activation energy, J·mol^−1^; k is the rate constant in mole fr.^−1^·min^−1^; A is the pre-exponential factor or Arrhenius factor in mole fr.^−1^·min^−1^; *R* is the gas constant, 8.314 J·mol^−1^·K^−1^; *T* is temperature in K.

The uncertainties *u* in the measured compositions (u(*x*)) were 0.005 (^1^H) and 0.001 (^19^F) mole fr. The sample weight (*m*) was measured with a Mass Comparator MC-1000 (A&D Company Ltd, Tokyo, Japan), which has a standard uncertainty of u(*m*) = ±0.0005 g. The temperature (*T*) in the stirred reactor was measured with mercury thermometers from Thermopribor OJSC (Moscow, Russia): 0⋯40–110 with a standard uncertainty u(*T*) = ±0.3 °C. The temperature measurement accuracy in the thermo-cabinet and NMR spectrometer is u(*T*) = 0.4 °C. For indirect values, the combined standard uncertainties σ are calculated using the uncertainty propagation based on the coefficient of determination and the standard deviations of the direct values included in their calculation. The procedure for calculating the combined standard uncertainties is presented in the Supplementary Materials File (Table S8).

## 4. Conclusions

The kinetics and chemical equilibrium of the esterification reaction of AAc and HFBol (using sulfuric acid as catalyst) were successfully determined experimentally. It is indicated that $K_{eq}$ depends significantly on the initial molar ratio of the reactants, catalyst concentration and temperature. $K_{eq}$ increases as $x_{HFBol}^{0}$ and/or $x_{H_{2}SO_{4}}$ and/or *T* increase. The esterification process of AAc and HFBol is characterized by a negative heat effect (${∆}_{r}H$ > 0). The total heat effect of the process will include the hydrophobic effects of the fluorinated components as well as the hydration of sulfuric and acetic acids due to the water formation in the system. The reaction system HFBol–AAc–HFBAc–water belongs to the homogeneous region of chemically equilibrium compositions at $x_{H_{2}SO_{4}}\leq 0.01$ mole fr. and $x_{HFBol}^{0}$ ≤ 0.35 mole fr.; at $x_{HFBol}^{0}$ ≥ 0.4 mole fr. and $x_{H_{2}SO_{4}}$ ≥ 0.0191 mole fr., it belongs to the heterogeneous region of chemically equilibrium compositions. The presence of a heterogeneous region is caused by the limited mutual solubility of water and HFBol and HFBAc. It has also been noticed that for initial molar ratio AAc/HFBol = 9/1 vs. 65/35 and $x_{H_{2}SO_{4}}=0.01$ mole fr., the dependences of the $K_{eq}$ on the temperature are characterized by a constant difference $∆K_{eq}$ = 0.027 ± 0.003, and the rate constant $k_{1}$ and the half-reaction time $\tau^{1/2}$ at constant temperature are independent of the initial molar ratio of the reactants within the experimental uncertainty. These observations can be extended to the entire homogeneous region of chemically equilibrium compositions with a high degree of confidence. For the homogeneous region of chemically equilibrium compositions, the dependences of the rate constant on temperature are also obtained, the parameters of Arrhenius equation are estimated, and the influence of the diffusion stage of the esterification reaction on the kinetic parameters of the process is shown.

The synthesis of HFBAc from AAc and HFBol seems to be a promising approach. In contrast to the synthesis by the transesterification reaction of isopropyl acetate and HFBol [20], in the present study, there are no side products. Moreover, in the investigated range of conditions, the esterification reaction is sufficiently fast. Thus, the reactive distillation process based on esterification seems to be feasible to product HFBAc. It should be noted that the technology readiness levels directly affect the cost of production, and the present work is one of the first to cover the technology of heptafluorobutyl acetate. So, for processes where heptafluorobutyl acetate is used in the production chain in one form or another, such studies should have a positive impact on the entire system.

In the context of future investigations, it is of interest from a theoretical point of view to carry out a comparative analysis of the influence of steric factors, acidity of functional groups and carbon chain halogenation on the kinetics, chemical equilibrium and thermodynamic of esterification of reaction systems with fluorinated and non-fluorinated reagents [16,21,22,23,24,25,26,27,28,29,30,31,32,33,34,35] under identical process conditions.

## Figures and Tables

**Table 1:** Dependence of the equilibrium constant $K_{eq}$ on the initial molar ratio of the reagents at atmospheric pressure, *T* = 50 °C and $x_{H_{2}SO_{4}}=0.01\pm 0.0007$ mole fr. calculated from Table S1 data.

$x_{HFBol}^{0}$	0.05	0.10	0.15	0.20	0.25	0.30	0.35	0.40	0.45
$x_{AAc}^{0}$	0.95	0.90	0.85	0.80	0.75	0.70	0.65	0.60	0.55
system type	homogeneous							heterogeneous	
$K_{eq}^{av}$	0.0625	0.0621	0.0810	0.0855	0.0904	0.0943	0.1050	0.1025	0.1237
σ($K_{eq}^{av}$)	0.0029	0.0052	0.0031	0.0034	0.0057	0.0063	0.0062	0.0095	0.0164
u(T) = ±0.4 °C									

**Table 2:** Dependence of the equilibrium constant $K_{eq}$ on the catalyst concentration at atmospheric pressure, initial molar ratio AAc/HFBol = 65/35 T = 30, 50 and 70 °C calculated from Table S2 data.

System Type			Homogeneous			Heterogeneous	
*T*, °C	1/(*T* + 273.15), K^−1^	$x_{H_{2}SO_{4}}$	0.0031	0.0056	0.0101	0.0191	0.0398
70	0.002914	$K_{eq}^{av}$	0.0994	0.1015	0.1120	0.1482	0.2531
		$\sigma (K_{eq}^{av})$	0.0077	0.0082	0.0081	0.0082	0.0314
		${lnK}_{eq}^{av}$	−2.3086	−2.2877	−2.1893	−1.9092	−1.3740
		$\sigma (lnK_{eq}^{av})$	0.077	0.081	0.072	0.055	0.124
50	0.003095	$K_{eq}^{av}$	0.0818	0.0828	0.0939	0.1252	0.2129
		$\sigma (K_{eq}^{av})$	0.0088	0.0089	0.0102	0.0134	0.0221
		${lnK}_{eq}^{av}$	−2.5035	−2.4913	−2.3655	−2.0778	−1.5469
		$\sigma (lnK_{eq}^{av})$	0.108	0.107	0.109	0.107	0.104
30	0.003299	$K_{eq}^{av}$	0.0633	0.0654	0.0761	0.0937	0.1712
		$\sigma (K_{eq}^{av})$	0.0021	0.0048	0.0066	0.0049	0.0097
		${lnK}_{eq}^{av}$	−2.7599	−2.7272	−2.5757	−2.3677	−1.7649
		$\sigma (lnK_{eq}^{av})$	0.033	0.073	0.087	0.052	0.057
*u*(*T*) = ±0.4 °C							

**Table 3:** Dependence of the equilibrium constant $K_{eq}$ on the temperature at initial molar ratio AAc/HFBol = 9/1 and 65/35, $x_{H_{2}SO_{4}}=0.01$ mole fr. and atmospheric pressure in temperature range from 30 to 90 °C calculated from the data in Tables S3 and S4.

*T*, °C	$K_{eq}^{av}$	$\sigma (K_{eq}^{av})$	${lnK}_{eq}^{av}$	$\sigma (lnK_{eq}^{av})$	1/(*T* + 273.15), K^−1^	*System Type*
*initial molar ratio AAc/HFBol = 9/1*						homogeneous
80	0.1000	0.0047	−2.3026	0.047	0.002832	
70	0.0849	0.0031	−2.4663	0.037	0.002914	
50	0.0621	0.0052	−2.7790	0.084	0.003095	
30	0.0528	0.0048	−2.9412	0.091	0.003299	
*initial molar ratio AAc/HFBol = 65/35*						
90	0.1383	0.0069	−1.9783	0.050	0.002754	
70	0.1120	0.0081	−2.1893	0.072	0.002914	
50	0.0939	0.0102	−2.3655	0.109	0.003095	
30	0.0761	0.0066	−2.5757	0.087	0.003299	
*u*(*T*) = ±0.3 °C						

**Table 4:** Van’t Hoff equation (Equation (15)) parameters for the esterification reaction of AAcand HFBol at $x_{H_{2}SO_{4}}=0.01$ mole fr., atmospheric pressure and temperature range from 30 to 90 °C calculated from Figure 3 and Figure 4.

$x_{H_{2}SO_{4}}$	*Initial Molar Ratio AAc*/*HFBol*	${∆}_{r}H$, kJ·mol^−1^	${∆}_{r}S$, J·mol^−1^·K^−1^	$K_{eq}=$	
0.01	9/1	11.418 ± 2.012	12.853 ± 6.113	$\begin{array}{c} \exp\left(\frac{-1373.38}{T}+1.5459\right) \end{array}$	(4)
	65/35	8.985 ± 1.825	8.163 ± 5.513	$\begin{array}{c} \exp\left(\frac{-1080.74}{T}+0.9818\right) \end{array}$	(5)
0.0031		9.761 ± 2.327	9.299 ± 7.230	$\begin{array}{c} \exp\left(\frac{-1173.99}{T}+1.1185\right) \end{array}$	(6)
0.0056		9.494 ± 2.899	8.653 ± 9.006	$\begin{array}{c} \exp\left(\frac{-1141.87}{T}+1.0408\right) \end{array}$	(7)
0.0191		9.942 ± 2.176	13.234 ± 6.762	$\begin{array}{c} \exp\left(\frac{-1195.76}{T}+1.5918\right) \end{array}$	(8)
0.0398		8.451 ± 3.466	13.234 ± 10.764	$\begin{array}{c} \exp\left(\frac{-1016.45}{T}+1.5918\right) \end{array}$	(9)
*T*—temperature in K					

**Table 5:** Dependence of the experimental rate constant $k_{1}$ of the esterification reaction of AAc and HFBol on the temperature *T* at initial molar ratio AAc/HFBol = 9/1, $x_{H_{2}SO_{4}}=0.01$ mole fr. and atmospheric pressure (laboratory stirred reactor) according to the Figure A1 data.

*T*, °C	$k_{1}^{av}$, mole fr.^−1^·min^−1^	*σ* $\left(k_{1}\right)$	$\ln k_{1}$	*σ* $\left(\ln k_{1}\right)$	1/(*T* + 273.15), K^−1^
40	0.002216	0.00005	−6.112	0.023	0.003193
50	0.003734	0.00005	−5.590	0.013	0.003095
60	0.006235	0.00021	−5.078	0.034	0.003002
70	0.013456	0.00039	−4.308	0.029	0.002914
80	0.022778	0.00133	−3.782	0.058	0.002832
90	0.032921	0.00263	−3.414	0.080	0.002754
*u*(*T*) = ±0.3 °C					

**Table 6:** Dependence of the experimental rate constant $k_{1}$ of the esterification reaction of AAc and HFBol on the temperature *T* at initial molar ratio AAc/HFBol = 9/1, $x_{H_{2}SO_{4}}=0.01$ mole fr. and atmospheric pressure (NMR apparatus) according to the Figure A2 data.

*T*, °C	$k_{1}^{av}$, mole fr.^−1^·min^−1^	*σ* $\left(k_{1}\right)$	$\ln k_{1}$	*σ* $\left(\ln k_{1}\right)$	1/(*T* + 273.15), K^−1^
40	0.001413	0.000035	−6.562	0.025	0.003193
45	0.002130	0.000274	−6.152	0.129	0.003143
50	0.002650	0.000532	−5.933	0.201	0.003095
55	0.003380	0.000016	−5.690	0.005	0.003047
60	0.003239	0.000047	−5.732	0.015	0.003002
65	0.004158	0.000021	−5.483	0.005	0.002957
70	0.005204	0.000065	−5.258	0.012	0.002914
*u*(*T*) = ±0.4 °C					

**Table 7:** Dependence of the experimental rate constant $k_{1}$ of the esterification reaction of AAc and HFBol on the temperature *T* at initial molar ratio AAc/HFBol = 65/35, $x_{H_{2}SO_{4}}=0.01$ mole fr. and atmospheric pressure (laboratory stirred reactor) according to the Figure A3 data.

*T*, °C	$k_{1}^{av}$, mole fr.^−1^·min^−1^	*σ* $\left(k_{1}\right)$	$\ln k_{1}$	*σ* $\left(\ln k_{1}\right)$	1/(*T* + 273.15), K^−1^
30	0.00121	0.00031	−6.717	0.256	0.003299
50	0.00319	0.00020	−5.748	0.063	0.003095
70	0.01141	0.00048	−4.473	0.042	0.002914
90	0.02734	0.00226	−3.599	0.083	0.002754
*u*(*T*) = ±0.3 °C					

**Table 8:** Dependence of the half-reaction time $\tau^{1/2}$ on the process conditions.

$x_{H_{2}SO_{4}}$	*Initial Molar Ratio AAc/HFBol*	*T,* °C	$K_{eq}^{\frac{AAc}{HFBol}=65/35}/K_{eq}^{\frac{AAc}{HFBol}=9/1}$	$\tau^{1/2},$ min
0.01	9/1	50	1.41	94.2
	65/35			92.6
	9/1	90	1.27	12.9
	65/35			12.6

**Table 9:** Arrhenius equation parameters (Equation (21)) for the esterification reaction of AAc and HFBol at $x_{H_{2}SO_{4}}=0.01$ mole fr. and atmospheric pressure calculated from Figure 6 data.

Initial Molar Ratio AAc/HFBol	Process Limiting Stage	$E_{a}$, J·mol^−1^	$u(E_{a})$, J·mol^−1^	*A*, mole fr.^−1^·min^−1^	*u*(*A*), mole fr.^−1^·min^−1^	$k_{1}$, mole fr.^−1^·min^−1^	
9/1 in NMR apparatus	Diffusion	34,919.4 *	2788.3	1075.8 *	1101.1	$\begin{array}{c} =exp\left(\frac{-4200.1}{T}+6.9808\right) \end{array}$	(10)
9/1 in laboratory stirred reactor	Reaction	53,249.3	2395.3	1,585,081	1,354,390	$\begin{array}{c} =exp\left(\frac{-6404.8}{T}+14.276\right) \end{array}$	(11)
65/35 in laboratory stirred reactor	Reaction	48,568.1	8348.4	263,414	797,825	$\begin{array}{c} =exp\left(\frac{-5841.7}{T}+12.481\right) \end{array}$	(12)
Merge 65/35 and 9/1 in laboratory stirred reactor	Reaction	51,042.2	5115.9	690,353	1,268,267	$\begin{array}{c} =exp\left(\frac{-6139.3}{T}+13.445\right) \end{array}$	(13)
* apparent value						*T*—temperature in K	

**Table 10:** Specifications of the compounds used.

Chemical Name	CAS-No	Molar Mass M, g·mol^−1^	Supplier	Initial Purity, Mass fr.	Purification in Laboratory	Mass fr. After Purification (GC ^a^)
HFBol	375-01-9	200.05	P&M Invest (Moscow, Russia)	0.60–0.90	Distillation, heteroazeotropic distillation	≥0.998
AAc	64-19-7	60.05	EKOS-1 (Moscow, Russia)	0.99	none	-
Sulfuric acid	7664-93-9	98.07	Merk (Rahway, NJ, USA)	0.98	none	-
DMSO-d6	2206-27-1	84.17	Solvex-D (Moscow, Russia)	0.998 atom % D	none	-

^a^ Gas chromatography–flame ionization detector.

## Data Availability

Data are contained within the article or Supplementary Materials.

## Supplementary Materials

The following supporting information can be downloaded at https://www.mdpi.com/article/10.3390/molecules30081744/s1, Table S1: Dependence of the composition of the reaction mixture at different initial molar ratios of the reagents on the time of thermostating at atmospheric pressure, $x_{H_{2}SO_{4}}=0.01$ mole fr. and T = 50 °C; Table S2: Dependence of the composition of the reaction mixture at different catalyst concentrations on the time of thermostating at atmospheric pressure, initial molar ratio AAc/HFBol = 65/35 and T = 30, 50 and 70 °C; Table S3: Dependence of the composition of the reaction mixture at different temperatures on the time of thermostating at atmospheric pressure, initial molar ratio AAc/HFBol = 9/1 and $x_{H_{2}SO_{4}}=0.01$ mole fr.; Table S4: Dependence of the composition of the reaction mixture at different temperatures on the time of thermostating at atmospheric pressure, initial molar ratio AAc/HFBol = 65/35, $x_{H_{2}SO_{4}}=0.01$ mole fr.; Table S5: Dependence of the composition of the reaction mixture at different temperatures on the time of thermostating at atmospheric pressure, initial molar ratio AAc/HFBol = 9/1, $x_{H_{2}SO_{4}}=0.01$ mole fr. obtained in laboratory stirred reactor; Table S6: Dependence of the composition of the reaction mixture at different temperatures on the time of thermostating at atmospheric pressure, initial molar ratio AAc/HFBol = 9/1, $x_{H_{2}SO_{4}}=0.01$ mole fr. obtained in NMR apparatus; Table S7: Dependence of the composition of the reaction mixture at different temperatures on the time of thermostating at atmospheric pressure, initial molar ratio AAc/HFBol = 65/35, $x_{H_{2}SO_{4}}=0.01$ mole fr. obtained in laboratory stirred reactor; Table S8: The procedure for calculating the combined standard uncertainties.

## Abbreviations

The following abbreviations are used in this manuscript:

A	pre-exponential factor or Arrhenius factor in mole fr.^−1^·min^−1^
*AAc*	acetic acid
$E_{a}$	activation energy, J·mol^−1^
*GC*	gas chromatography
$\Delta_{r}H$	standard enthalpy of the reaction, J·mol^−1^
*HFBAc*	2,2,3,3,4,4,4-heptafluorobutyl acetate
*HFBol*	2,2,3,3,4,4,4-heptafluorobutan-1-ol
$K_{eq}$	chemical equilibrium constant
$k_{1}$	rate constant of the forward reaction in mole fr.^−1^·min^−1^
$k_{2}$	rate constant of the reverse reaction in mole fr.^−1^·min^−1^
*M*	the molar mass in g·mol^−1^
*m*	sample weight in g
*NMR*	nuclear magnetic resonance
*n*	number of parallel experiments
*R*	gas constant, 8.314 J·mol^−1^·K^−1^
$\Delta_{r}S$	standard entropy of the reaction, J·mol^−1^·K^−1^
*T*	temperature in K or in °C
*u*	standard uncertainty
v	reaction rate in mol min^−1^
$x_{i}$	mole fraction of component *i*
$x_{i}^{0}$	the mole fraction of component *i* at *τ* = 0
σ	combined standard uncertainty
$\tau^{1/2}$	half-reaction time in min
*τ*	reaction time in min or hour or day
indexes	
*av*	the average value
*i*	component index
