# Supplementary material for: 2,2,3,3,4,4,4-Heptafluorobutyl Acetate—Chemical Equilibrium and Kinetics of the Esterification Reaction of 2,2,3,3,4,4,4-Heptafluorobutan-1-ol and Acetic Acid in the Presence of an Acidic Catalyst
Source: Molecules. 2025 Apr 13;30(8):1744. doi: 10.3390/molecules30081744 (PMC12029711; doi:10.3390/molecules30081744)
Supplement: Supplementary file 1 [file molecules-30-01744-s001.zip › molecules-3564767-supplementary.pdf]

Supplementary data

Chemical equilibrium data

<u>Dependence of the equilibrium constant on the initial molar ratio of the reagents</u>

Table S1: Dependence of the composition of the reaction mixture at different initial molar ratios of the reagents on the time of thermostating at atmospheric pressure, $x_{H_2SO_4} = 0.01$ mole fr. and T = 50 °C

τ, days	x_{AAc}	x_{HFBol}	x_{HFBAc}	x_{H_2O}	τ, days	x_{AAc}	x_{HFBol}	x_{HFBAc}	x_{H_2O}
**Initial molar ratio AAc/HFBol=95/5, $x_{H_2SO_4} = 0.0102$ mole fr., K_{eq}^{av}= 0.0625**					**Initial molar ratio AAc/HFBol=85/15, $x_{H_2SO_4} = 0.0103$ mole fr., K_{eq}^{av}= 0.0810**				
0	0.9501	0.0499	0	0	0	0.8499	0.1501	0	0
2	0.9201	0.0201	0.0299	0.0299	2	0.7913	0.0915	0.0586	0.0586
9	0.9183	0.0183	0.0317	0.0317	9	0.7823	0.0825	0.0676	0.0676
16	0.9175	0.0175	0.0325	0.0325	16	0.7839	0.0841	0.0660	0.0660
23	0.9172	0.0172	0.0328	0.0328	23	0.7777	0.0779	0.0722	0.0722
30	0.9179	0.0179	0.0321	0.0321	30	0.7795	0.0797	0.0704	0.0704
37	0.9179	0.0179	0.0321	0.0321	37	0.7792	0.0794	0.0707	0.0707
44	0.9176	0.0176	0.0324	0.0324	44	0.7787	0.0789	0.0712	0.0712
51	0.9182	0.0180	0.0319	0.0319	51	0.7801	0.0803	0.0698	0.0698
58	0.9187	0.0185	0.0314	0.0314	58	0.7778	0.0780	0.0721	0.0721
65	0.9187	0.0185	0.0314	0.0314	65	0.7809	0.0811	0.0690	0.0690
**Initial molar ratio AAc/HFBol=80/20, $x_{H_2SO_4} = 0.0101$ mole fr., K_{eq}^{av}= 0.0855**					**Initial molar ratio AAc/HFBol=75/25, $x_{H_2SO_4} = 0.0118$ mole fr., K_{eq}^{av}= 0.0904**				
0	0.7999	0.2001	0	0	0	0.7484	0.2516	0	0
2	0.7378	0.1380	0.0621	0.0621	2	0.6847	0.1879	0.0637	0.0637
9	0.7226	0.1228	0.0773	0.0773	9	0.6663	0.1695	0.0821	0.0821
16	0.7160	0.1162	0.0839	0.0839	16	0.6595	0.1627	0.0888	0.0888
23	0.7174	0.1176	0.0825	0.0825	23	0.6546	0.1580	0.0937	0.0937
30	0.7153	0.1155	0.0846	0.0846	30	0.6535	0.1567	0.0949	0.0949
37	0.7137	0.1139	0.0862	0.0862	37	0.6533	0.1565	0.0951	0.0951
44	0.7134	0.1136	0.0865	0.0865	44	0.6478	0.1510	0.1006	0.1006
51	0.7169	0.1171	0.0830	0.0830	51	0.6564	0.1596	0.0920	0.0920
58	0.7157	0.1159	0.0842	0.0842	58	0.6499	0.1533	0.0984	0.0984
65	0.7171	0.1173	0.0828	0.0828	65	0.6526	0.1558	0.0958	0.0958
**Initial molar ratio AAc/HFBol=70/30, $x_{H_2SO_4} = 0.0111$ mole fr., K_{eq}^{av}= 0.0943**					**Initial molar ratio AAc/HFBol=65/35, $x_{H_2SO_4} = 0.0098$ mole fr., K_{eq}^{av}= 0.1050**				
0	0.7005	0.2995	0	0	0	0.6476	0.3524	0	0
2	0.6501	0.2489	0.0505	0.0505	2	0.5343	0.2391	0.1133	0.1133
9	0.6123	0.2111	0.0883	0.0883	9	0.5308	0.2354	0.1169	0.1169
16	0.6036	0.2024	0.0970	0.0970	16	0.5374	0.2420	0.1103	0.1103
23	0.5915	0.1903	0.1091	0.1091	23	0.5307	0.2355	0.1169	0.1169
30	0.5964	0.1954	0.1041	0.1041	30	0.5296	0.2344	0.1180	0.1180
37	0.5965	0.1955	0.1040	0.1040	37	0.5328	0.2373	0.1150	0.1150
44	0.5921	0.1911	0.1084	0.1084	44	0.5389	0.2437	0.1087	0.1087
51	0.6030	0.2020	0.0975	0.0975	51	0.5282	0.2328	0.1195	0.1195
58	0.5955	0.1945	0.1050	0.1050	58	0.5320	0.2366	0.1157	0.1157
65	0.5972	0.1962	0.1033	0.1033	65	0.5314	0.2362	0.1162	0.1162
**Initial molar ratio AAc/HFBol=60/40, $x_{H_2SO_4} = 0.0111$ mole fr., K_{eq}^{av}= 0.1025**					**Initial molar ratio AAc/HFBol=55/45, $x_{H_2SO_4} = 0.0110$ mole fr., K_{eq}^{av}= 0.1237**				
0	0.6476	0.3524	0	0	0	0.5277	0.4723	0	0
2	0.5343	0.2391	0.1133	0.1133	2	0.4276	0.3722	0.1001	0.1001
9	0.5308	0.2354	0.1169	0.1169	9	0.3986	0.3432	0.1291	0.1291
16	0.5374	0.2420	0.1103	0.1103	16	0.3918	0.3366	0.1358	0.1358
23	0.5307	0.2355	0.1169	0.1169	23	0.3487	0.2933	0.1790	0.1790
30	0.5296	0.2344	0.1180	0.1180	30	0.3992	0.3438	0.1285	0.1285
37	0.5326	0.2374	0.1150	0.1150	37	0.3963	0.3409	0.1314	0.1314
44	0.5389	0.2437	0.1087	0.1087	44	0.4031	0.3477	0.1246	0.1246
51	0.5282	0.2328	0.1195	0.1195	51	0.3968	0.3416	0.1308	0.1308
58	0.5320	0.2366	0.1157	0.1157	58	0.4042	0.3488	0.1235	0.1235

65	0.5314	0.2362	0.1162	0.1162	65	0.3939	0.3385	0.1338	0.1338

u(x_i) = 0.005 mole fr.; u(T) = 0.4 ºC; Initial molar ratio AAc/HFBol=90/10 is given in Table S2

Dependence of the equilibrium constant on the catalyst concentration

Table S2: Dependence of the composition of the reaction mixture at different catalyst concentrations on the time of thermostating at atmospheric pressure, initial molar ratio AAc/HFBol = 65/35 and T = 30, 50 and 70 °C

τ, days	x_{AAc}	x_{HFBol}	x_{HFBAc}	x_{H_2O}	τ, days	x_{AAc}	x_{HFBol}	x_{HFBAc}	x_{H_2O}
				**$x_{H_2SO_4} = 0.0031$ mole fr., $K_{eq}^{av} = 0.0994$, T = 70 ºC**					
		Set No. 1					***Set No. 2***		
7	0.5346	0.2346	0.1154	0.1154	7	0.5367	0.2367	0.1133	0.1133
14	0.5252	0.2252	0.1248	0.1248	14	0.5533	0.2533	0.0967	0.0967
21	0.5373	0.2373	0.1127	0.1127	21	0.5422	0.2422	0.1078	0.1078
28	0.5376	0.2376	0.1124	0.1124	28	0.5374	0.2374	0.1126	0.1126
35	0.5371	0.2371	0.1129	0.1129	35	0.5383	0.2383	0.1117	0.1117
				Set No. 3					
7	0.5408	0.2408	0.1092	0.1092	28	0.5366	0.2366	0.1134	0.1134
14	0.5397	0.2397	0.1103	0.1103	35	0.5361	0.2361	0.1139	0.1139
21	0.5325	0.2325	0.1175	0.1175					
				**$x_{H_2SO_4} = 0.0056$ mole fr., $K_{eq}^{av} = 0.1015$, T = 70 ºC**					
		Set No. 1					***Set No. 2***		
7	0.5379	0.2379	0.1121	0.1121	7	0.5281	0.2281	0.1219	0.1219
14	0.5518	0.2518	0.0982	0.0982	14	0.5387	0.2387	0.1113	0.1113
21	0.5332	0.2332	0.1168	0.1168	21	0.5412	0.2412	0.1088	0.1088
28	0.5404	0.2404	0.1096	0.1096	28	0.5383	0.2383	0.1117	0.1117
35	0.5359	0.2359	0.1141	0.1141	35	0.5374	0.2374	0.1126	0.1126
				Set No. 3					
7	0.5304	0.2304	0.1196	0.1196	28	0.5315	0.2315	0.1185	0.1185
14	0.5393	0.2393	0.1107	0.1107	35	0.5352	0.2352	0.1148	0.1148
21	0.5322	0.2322	0.1178	0.1178					
				**$x_{H_2SO_4} = 0.0191$ mole fr., $K_{eq}^{av} = 0.1482$, T = 70 ºC**					
		Set No. 1					***Set No. 2***		
7	0.5188	0.2188	0.1312	0.1312	7	0.5118	0.2118	0.1382	0.1382
14	0.5193	0.2193	0.1307	0.1307	14	0.5209	0.2209	0.1291	0.1291
21	0.5230	0.2230	0.1270	0.1270	21	0.5243	0.2243	0.1257	0.1257
28	0.5219	0.2219	0.1281	0.1281	28	0.5150	0.2150	0.1350	0.1350
35	0.5197	0.2197	0.1303	0.1303	35	0.5225	0.2225	0.1275	0.1275
				Set No. 3					
7	0.5175	0.2175	0.1325	0.1325	28	0.5227	0.2227	0.1273	0.1273
14	0.5251	0.2251	0.1249	0.1249	35	0.5203	0.2203	0.1297	0.1297
21	0.5166	0.2166	0.1334	0.1334					
				**$x_{H_2SO_4} = 0.0398$ mole fr., $K_{eq}^{av} = 0.2531$, T = 70 ºC**					
		Set No. 1					***Set No. 2***		
7	0.4869	0.1869	0.1631	0.1631	7	0.4971	0.1971	0.1529	0.1529
14	0.4985	0.1985	0.1515	0.1515	14	0.5039	0.2039	0.1461	0.1461
21	0.4888	0.1888	0.1612	0.1612	21	0.4992	0.1992	0.1508	0.1508
28	0.4881	0.1881	0.1619	0.1619	28	0.4980	0.1980	0.1520	0.1520
35	0.4972	0.1972	0.1528	0.1528	35	0.5014	0.2014	0.1486	0.1486
				Set No. 3					
7	0.4928	0.1928	0.1572	0.1572	28	0.5108	0.2108	0.1392	0.1392
14	0.4822	0.1822	0.1678	0.1678	35	0.4835	0.1835	0.1665	0.1665
21	0.4916	0.1916	0.1584	0.1584					
				**$x_{H_2SO_4} = 0.0031$ mole fr., $K_{eq}^{av} = 0.0818$, T = 50 ºC**					
		Set No. 1					***Set No. 2***		
3	0.5454	0.2454	0.1046	0.1046	3	0.5504	0.2504	0.0996	0.0996
10	0.5478	0.2478	0.1022	0.1022	10	0.5346	0.2346	0.1154	0.1154
18	0.5581	0.2581	0.0919	0.0919	18	0.5349	0.2349	0.1151	0.1151
24	0.5453	0.2453	0.1047	0.1047	24	0.5489	0.2489	0.1011	0.1011
31	0.5451	0.2451	0.1049	0.1049	31	0.5465	0.2465	0.1035	0.1035
38	0.5503	0.2503	0.0997	0.0997	38	0.5479	0.2479	0.1021	0.1021
46	0.5454	0.2454	0.1046	0.1046	46	0.5485	0.2485	0.1015	0.1015
52	0.5382	0.2382	0.1118	0.1118	52	0.5399	0.2399	0.1101	0.1101
				Set No. 3					

3	0.5374	0.2374	0.1126	0.1126	31	0.5469	0.2469	0.1031	0.1031
10	0.5418	0.2418	0.1082	0.1082	38	0.5465	0.2465	0.1035	0.1035
18	0.5503	0.2503	0.0997	0.0997	46	0.5500	0.2500	0.1000	0.1000
24	0.5385	0.2385	0.1115	0.1115	52	0.5582	0.2582	0.0918	0.0918

$x_{H_2SO_4} = \mathbf{0.0056}$ **mole fr.**, $K_{eq}^{av} = \mathbf{0.0828}$, *T* = 50 ºC

		Set No. 1					*Set No. 2*		
3	0.5357	0.2357	0.1143	0.1143	3	0.5395	0.2395	0.1105	0.1105
10	0.5418	0.2418	0.1082	0.1082	10	0.5396	0.2396	0.1104	0.1104
18	0.5461	0.2461	0.1039	0.1039	18	0.5491	0.2491	0.1009	0.1009
24	0.5606	0.2606	0.0894	0.0894	24	0.5318	0.2318	0.1182	0.1182
31	0.5489	0.2489	0.1011	0.1011	31	0.5486	0.2486	0.1014	0.1014
38	0.5343	0.2343	0.1157	0.1157	38	0.5468	0.2468	0.1032	0.1032
46	0.5440	0.2440	0.1060	0.1060	46	0.5506	0.2506	0.0994	0.0994
52	0.5417	0.2417	0.1083	0.1083	52	0.5451	0.2451	0.1049	0.1049
				Set No. 3					
3	0.5484	0.2484	0.1016	0.1016	31	0.5494	0.2494	0.1006	0.1006
10	0.5402	0.2402	0.1098	0.1098	38	0.5484	0.2484	0.1016	0.1016
18	0.5594	0.2594	0.0906	0.0906	46	0.5460	0.2460	0.1040	0.1040
24	0.5561	0.2561	0.0939	0.0939	52	0.5362	0.2362	0.1138	0.1138

$x_{H_2SO_4} = \mathbf{0.0191}$ **mole fr.**, $K_{eq}^{av} = \mathbf{0.1252}$, *T* = 50 ºC

		Set No. 1					*Set No. 2*		
3	0.5175	0.2175	0.1325	0.1325	3	0.5326	0.2326	0.1174	0.1174
10	0.5298	0.2298	0.1202	0.1202	10	0.5335	0.2335	0.1165	0.1165
18	0.5185	0.2185	0.1315	0.1315	18	0.5347	0.2347	0.1153	0.1153
24	0.5190	0.2190	0.1310	0.1310	24	0.5301	0.2301	0.1199	0.1199
31	0.5448	0.2448	0.1052	0.1052	31	0.5319	0.2319	0.1181	0.1181
38	0.5324	0.2324	0.1176	0.1176	38	0.5327	0.2327	0.1173	0.1173
46	0.5164	0.2164	0.1336	0.1336	46	0.5305	0.2305	0.1195	0.1195
52	0.5376	0.2376	0.1124	0.1124	52	0.5189	0.2189	0.1311	0.1311
				Set No. 3					
3	0.5174	0.2174	0.1326	0.1326	31	0.5335	0.2335	0.1165	0.1165
10	0.5321	0.2321	0.1179	0.1179	38	0.5330	0.2330	0.1170	0.1170
18	0.5181	0.2181	0.1319	0.1319	46	0.5188	0.2188	0.1312	0.1312
24	0.5266	0.2266	0.1234	0.1234	52	0.5310	0.2310	0.1190	0.1190

$x_{H_2SO_4} = \mathbf{0.0398}$ **mole fr.**, $K_{eq}^{av} = \mathbf{0.2129}$, *T* = 50 ºC

		Set No. 1					*Set No. 2*		
3	0.5072	0.2072	0.1428	0.1428	3	0.5200	0.2200	0.1300	0.1300
10	0.4986	0.1986	0.1514	0.1514	10	0.4998	0.1998	0.1502	0.1502
18	0.5054	0.2054	0.1446	0.1446	18	0.5073	0.2073	0.1427	0.1427
24	0.5018	0.2018	0.1482	0.1482	24	0.4882	0.1882	0.1618	0.1618
31	0.5045	0.2045	0.1455	0.1455	31	0.5151	0.2151	0.1349	0.1349
38	0.5067	0.2067	0.1433	0.1433	38	0.4945	0.1945	0.1555	0.1555
46	0.5061	0.2061	0.1439	0.1439	46	0.5043	0.2043	0.1457	0.1457
52	0.4949	0.1949	0.1551	0.1551	52	0.4944	0.1944	0.1556	0.1556
				Set No. 3					
3	0.5081	0.2081	0.1419	0.1419	31	0.5049	0.2049	0.1451	0.1451
10	0.5036	0.2036	0.1464	0.1464	38	0.5103	0.2103	0.1397	0.1397
18	0.5061	0.2061	0.1439	0.1439	46	0.5070	0.2070	0.1430	0.1430
24	0.4975	0.1975	0.1525	0.1525	52	0.4900	0.1900	0.1600	0.1600

$x_{H_2SO_4} = \mathbf{0.0031}$ **mole fr.**, $K_{eq}^{av} = \mathbf{0.0633}$, *T* = 30 ºC

		Set No. 1					*Set No. 2*		
3	0.5720	0.2720	0.0780	0.0780	3	0.5767	0.2767	0.0733	0.0733
10	0.5518	0.2518	0.0982	0.0982	10	0.5541	0.2541	0.0959	0.0959
18	0.5581	0.2581	0.0919	0.0919	18	0.5543	0.2543	0.0957	0.0957
24	0.5519	0.2519	0.0981	0.0981	24	0.5544	0.2544	0.0956	0.0956
31	0.5546	0.2546	0.0954	0.0954	31	0.5559	0.2559	0.0941	0.0941
38	0.5551	0.2551	0.0949	0.0949	38	0.5548	0.2548	0.0952	0.0952
46	0.5683	0.2683	0.0817	0.0817	46	0.5542	0.2542	0.0958	0.0958
52	0.5680	0.2680	0.0820	0.0820	52	0.5429	0.2429	0.1071	0.1071
				Set No. 3					
3	0.5736	0.2736	0.0764	0.0764	31	0.5551	0.2551	0.0949	0.0949
10	0.5604	0.2604	0.0896	0.0896	38	0.5565	0.2565	0.0935	0.0935
18	0.5505	0.2505	0.0995	0.0995	46	0.5543	0.2543	0.0957	0.0957
24	0.5582	0.2582	0.0918	0.0918	52	0.5535	0.2535	0.0965	0.0965

$x_{H_2SO_4} = \mathbf{0.0056}$ **mole fr.**, $K_{eq}^{av} = \mathbf{0.0654}$, *T* = 30 ºC

		Set No. 1					*Set No. 2*		

3	0.5577	0.2577	0.0923	0.0923	3	0.5675	0.2675	0.0825	0.0825
10	0.5511	0.2511	0.0989	0.0989	10	0.5573	0.2573	0.0927	0.0927
18	0.5491	0.2491	0.1009	0.1009	18	0.5511	0.2511	0.0989	0.0989
24	0.5489	0.2489	0.1011	0.1011	24	0.5518	0.2518	0.0982	0.0982
31	0.5544	0.2544	0.0956	0.0956	31	0.5567	0.2567	0.0933	0.0933
38	0.5564	0.2564	0.0936	0.0936	38	0.5550	0.2550	0.0950	0.0950
46	0.5487	0.2487	0.1013	0.1013	46	0.5547	0.2547	0.0953	0.0953
52	0.5595	0.2595	0.0905	0.0905	52	0.5532	0.2532	0.0968	0.0968
				Set No. 3					
3	0.5659	0.2659	0.0841	0.0841	31	0.5664	0.2664	0.0836	0.0836
10	0.5602	0.2602	0.0898	0.0898	38	0.5548	0.2548	0.0952	0.0952
18	0.5553	0.2553	0.0947	0.0947	46	0.5541	0.2541	0.0959	0.0959
24	0.5486	0.2486	0.1014	0.1014	52	0.5514	0.2514	0.0986	0.0986
				$x_{H_2SO_4} = \mathbf{0.0191}$ **mole fr.,** $K_{eq}^{av} = \mathbf{0.0937}$, T = 30 ºC					
		Set No. 1					*Set No. 2*		
3	0.5435	0.2435	0.1065	0.1065	3	0.5439	0.2439	0.1061	0.1061
10	0.5340	0.2340	0.1160	0.1160	10	0.5362	0.2362	0.1138	0.1138
18	0.5411	0.2411	0.1089	0.1089	18	0.5389	0.2389	0.1111	0.1111
24	0.5310	0.2310	0.1190	0.1190	24	0.5371	0.2371	0.1129	0.1129
31	0.5396	0.2396	0.1104	0.1104	31	0.5399	0.2399	0.1101	0.1101
38	0.5386	0.2386	0.1114	0.1114	38	0.5430	0.2430	0.1070	0.1070
46	0.5508	0.2508	0.0992	0.0992	46	0.5396	0.2396	0.1104	0.1104
52	0.5396	0.2396	0.1104	0.1104	52	0.5575	0.2575	0.0925	0.0925
				Set No. 3					
3	0.5393	0.2393	0.1107	0.1107	31	0.5413	0.2413	0.1087	0.1087
10	0.5340	0.2340	0.1160	0.1160	38	0.5397	0.2397	0.1103	0.1103
18	0.5438	0.2438	0.1062	0.1062	46	0.5330	0.2330	0.1170	0.1170
24	0.5389	0.2389	0.1111	0.1111	52	0.5385	0.2385	0.1115	0.1115
				$x_{H_2SO_4} = \mathbf{0.0398}$ **mole fr.,** $K_{eq}^{av} = \mathbf{0.1712}$, T = 30 ºC					
		Set No. 1					*Set No. 2*		
3	0.5129	0.2129	0.1371	0.1371	3	0.5163	0.2163	0.1337	0.1337
10	0.5171	0.2171	0.1329	0.1329	10	0.5126	0.2126	0.1374	0.1374
18	0.5154	0.2154	0.1346	0.1346	18	0.5156	0.2156	0.1344	0.1344
24	0.5060	0.2060	0.1440	0.1440	24	0.5101	0.2101	0.1399	0.1399
31	0.5118	0.2118	0.1382	0.1382	31	0.5135	0.2135	0.1365	0.1365
38	0.5141	0.2141	0.1359	0.1359	38	0.5180	0.2180	0.1320	0.1320
46	0.5140	0.2140	0.1360	0.1360	46	0.5136	0.2136	0.1364	0.1364
52	0.5141	0.2141	0.1359	0.1359	52	0.5112	0.2112	0.1388	0.1388
				Set No. 3					
3	0.5172	0.2172	0.1328	0.1328	31	0.5235	0.2235	0.1265	0.1265
10	0.5054	0.2054	0.1446	0.1446	38	0.5178	0.2178	0.1322	0.1322
18	0.5136	0.2136	0.1364	0.1364	46	0.5036	0.2036	0.1464	0.1464
24	0.5123	0.2123	0.1377	0.1377	52	0.5100	0.2100	0.1400	0.1400

u(x_i) = 0.005 mole fr.; u(T) = 0.4 ºC; $x_{H_2SO_4}$ = 0.01 mole fr. at T = 30, 50 and 70 ºC is given in Table S4

Dependence of the equilibrium constant on temperature

Table S3: Dependence of the composition of the reaction mixture at different temperatures on the time of thermostating at atmospheric pressure, initial molar ratio AAc/HFBol = 9/1 and $x_{H_2SO_4} = 0.01$ mole fr.

τ, days	x_{AAc}	x_{HFBol}	x_{HFBAc}	x_{H_2O}	τ, days	x_{AAc}	x_{HFBol}	x_{HFBAc}	x_{H_2O}
				T = 30 ºC, $x_{H_2SO_4} = \mathbf{0.0096}$ **mole fr.,** $K_{eq}^{av} = \mathbf{0.0528}$					
		Set No. 1					*Set No. 2*		
17	0.8536	0.0536	0.0464	0.0464	17	0.8490	0.0490	0.051	0.051
24	0.8519	0.0519	0.0481	0.0481	24	0.8506	0.0506	0.0494	0.0494
31	0.8514	0.0514	0.0486	0.0486	31	0.8487	0.0487	0.0513	0.0513
39	0.8523	0.0523	0.0477	0.0477	39	0.8511	0.0511	0.0489	0.0489
45	0.8522	0.0522	0.0478	0.0478	45	0.8508	0.0508	0.0492	0.0492
52	0.8559	0.0559	0.0441	0.0441	52	0.8509	0.0509	0.0491	0.0491
59	0.8535	0.0535	0.0465	0.0465	59	0.8521	0.0521	0.0479	0.0479
67	0.8530	0.0530	0.0470	0.0470	67	0.8548	0.0548	0.0452	0.0452
73	0.8519	0.0519	0.0481	0.0481	73	0.8556	0.0556	0.0444	0.0444
		Set No. 3					*Set No. 4*		
17	0.8511	0.0511	0.0489	0.0489	17	0.8515	0.0515	0.0485	0.0485

24	0.8499	0.0499	0.0501	0.0501	24	0.8512	0.0512	0.0488	0.0488
31	0.8480	0.0480	0.0520	0.0520	31	0.8513	0.0513	0.0487	0.0487
39	0.8513	0.0513	0.0487	0.0487	39	0.8523	0.0523	0.0477	0.0477
45	0.8509	0.0509	0.0491	0.0491	45	0.8497	0.0497	0.0503	0.0503
52	0.8511	0.0511	0.0489	0.0489	52	0.8555	0.0555	0.0445	0.0445
59	0.8487	0.0487	0.0513	0.0513	59	0.8509	0.0509	0.0491	0.0491
67	0.8563	0.0563	0.0437	0.0437	67	0.8524	0.0524	0.0476	0.0476
73	0.8504	0.0504	0.0496	0.0496	73	0.8561	0.0561	0.0439	0.0439
**T = 50 ºC, $x_{H_2SO_4} = \mathbf{0.0096}$ mole fr., $K_{eq}^{av} = \mathbf{0.0621}$**									
		Set No. 1					***Set No. 2***		
7	0.8495	0.0495	0.0505	0.0505	7	0.8521	0.0521	0.0479	0.0479
14	0.8486	0.0486	0.0514	0.0514	14	0.8509	0.0509	0.0491	0.0491
21	0.8503	0.0503	0.0497	0.0497	21	0.8528	0.0528	0.0472	0.0472
28	0.8486	0.0486	0.0514	0.0514	28	0.8483	0.0483	0.0517	0.0517
35	0.854	0.054	0.046	0.046	35	0.8492	0.0492	0.0508	0.0508
42	0.8502	0.0502	0.0498	0.0498	42	0.8523	0.0523	0.0477	0.0477
49	0.8480	0.0480	0.0520	0.0520	49	0.8466	0.0534	0.0534	0.0534
		Set No. 3					***Set No. 4***		
7	0.8493	0.0493	0.0507	0.0507	7	0.8548	0.0548	0.0452	0.0452
14	0.8458	0.0458	0.0542	0.0542	14	0.8503	0.0503	0.0497	0.0497
21	0.8496	0.0496	0.0504	0.0504	21	0.8503	0.0503	0.0497	0.0497
28	0.8487	0.0487	0.0513	0.0513	28	0.8516	0.0516	0.0484	0.0484
35	0.8437	0.0437	0.0563	0.0563	35	0.8510	0.0510	0.0490	0.0490
42	0.8505	0.0505	0.0495	0.0495	42	0.8503	0.0503	0.0497	0.0497
49	0.8467	0.0467	0.0533	0.0533	49	0.8475	0.0475	0.0525	0.0525
**T = 70 ºC, $x_{H_2SO_4} = \mathbf{0.0095}$ mole fr., $K_{eq}^{av} = \mathbf{0.0849}$**					**T = 80 ºC, $x_{H_2SO_4} = \mathbf{0.0101}$ mole fr., $K_{eq}^{av} = \mathbf{0.1000}$**				
0	0.8998	0.1002	0.0000	0.0000	**τ, min**	x_{AAc}	x_{HFBol}	x_{HFBAc}	x_{H_2O}
6	0.8424	0.0428	0.0574	0.0574	90	0.8410	0.0416	0.0587	0.0587
13	0.8437	0.0441	0.0561	0.0561	120	0.8402	0.0408	0.0595	0.0595
34	0.8445	0.0449	0.0553	0.0553	150	0.8404	0.0410	0.0593	0.0593
41	0.8440	0.0444	0.0558	0.0558	180	0.8402	0.0408	0.0595	0.0595
					211	0.8399	0.0405	0.0598	0.0598
					240	0.8430	0.0436	0.0567	0.0567

u(x_i) = 0.005 mole fr.; u(T) = 0.4 ºC [30 ºC; 70 ºC]; u(T) = 0.3 ºC [80 ºC]

Table S4: Dependence of the composition of the reaction mixture at different temperatures on the time of thermostating at atmospheric pressure, initial molar ratio AAc/HFBol = 65/35, $x_{H_2SO_4}$ = 0.01 mole fr.

τ, days	x_{AAc}	x_{HFBol}	x_{HFBAc}	x_{H_2O}	τ, days	x_{AAc}	x_{HFBol}	x_{HFBAc}	x_{H_2O}
**T = 30 ºC, $x_{H_2SO_4} = \mathbf{0.0101}$ mole fr., K_{eq}^{av}= 0.0761**									
		Set No. 1					***Set No. 2***		
3	0.5511	0.2511	0.0989	0.0989	3	0.5424	0.2424	0.1076	0.1076
10	0.5389	0.2389	0.1111	0.1111	10	0.544	0.244	0.106	0.106
18	0.5436	0.2436	0.1064	0.1064	18	0.5454	0.2454	0.1046	0.1046
24	0.5472	0.2472	0.1028	0.1028	24	0.5501	0.2501	0.0999	0.0999
31	0.5512	0.2512	0.0988	0.0988	31	0.5602	0.2602	0.0898	0.0898
38	0.5528	0.2528	0.0972	0.0972	38	0.5481	0.2481	0.1019	0.1019
46	0.5510	0.2510	0.0990	0.0990	46	0.5500	0.2500	0.1000	0.1000
52	0.5514	0.2514	0.0986	0.0986	52	0.5332	0.2332	0.1168	0.1168
				Set No. 3					
3	0.5500	0.2500	0.1000	0.1000	31	0.5509	0.2509	0.0991	0.0991
10	0.5429	0.2429	0.1071	0.1071	38	0.5489	0.2489	0.1011	0.1011
18	0.5447	0.2447	0.1053	0.1053	46	0.5608	0.2608	0.0892	0.0892
24	0.5441	0.2441	0.1059	0.1059	52	0.5330	0.2330	0.1170	0.1170
**T = 50 ºC, $x_{H_2SO_4} = \mathbf{0.0101}$ mole fr., K_{eq}^{av}= 0.0939**									
		Set No. 1					***Set No. 2***		
7	0.5414	0.2414	0.1086	0.1086	7	0.5412	0.2412	0.1088	0.1088
12	0.5391	0.2391	0.1109	0.1109	12	0.5414	0.2414	0.1086	0.1086
19	0.5442	0.2442	0.1058	0.1058	19	0.5286	0.2286	0.1214	0.1214
26	0.5527	0.2527	0.0973	0.0973	26	0.5432	0.2432	0.1068	0.1068
33	0.5420	0.2420	0.1080	0.1080	33	0.5270	0.2270	0.1230	0.1230
40	0.5449	0.2449	0.1051	0.1051	40	0.5537	0.2537	0.0963	0.0963
47	0.5411	0.2411	0.1089	0.1089	47	0.5287	0.2287	0.1213	0.1213
54	0.5330	0.2330	0.1170	0.1170	54	0.5333	0.2333	0.1167	0.1167

				Set No. 3					
7	0.5542	0.2542	0.0958	0.0958	33	0.5284	0.2284	0.1216	0.1216
12	0.5382	0.2382	0.1118	0.1118	40	0.5535	0.2535	0.0965	0.0965
19	0.5429	0.2429	0.1071	0.1071	47	0.5337	0.2337	0.1163	0.1163
26	0.5406	0.2406	0.1094	0.1094	54	0.5407	0.2407	0.1093	0.1093
				**T = 70 ºC, $x_{H_2SO_4}$ = 0.0101 mole fr., K_{eq}^{av}= 0.1120**					
		Set No. 1					*Set No. 2*		
7	0.5389	0.2389	0.1111	0.1111	7	0.5291	0.2291	0.1209	0.1209
14	0.5354	0.2354	0.1146	0.1146	14	0.5350	0.2350	0.1150	0.1150
21	0.5369	0.2369	0.1131	0.1131	21	0.5306	0.2306	0.1194	0.1194
28	0.5341	0.2341	0.1159	0.1159	28	0.5339	0.2339	0.1161	0.1161
35	0.5333	0.2333	0.1167	0.1167	35	0.5316	0.2316	0.1184	0.1184
				Set No. 3					
7	0.5297	0.2297	0.1203	0.1203	28	0.5277	0.2277	0.1223	0.1223
14	0.5233	0.2233	0.1267	0.1267	35	0.5340	0.2340	0.1160	0.1160
21	0.5335	0.2335	0.1165	0.1165					
				**T = 90 ºC, $x_{H_2SO_4}$ = 0.0101 mole fr., K_{eq}^{av}= 0.1383**					
3	0.5422	0.2220	0.1179	0.1179	18	0.5065	0.2335	0.1300	0.1300
5	0.5252	0.2252	0.1248	0.1248	19	0.5067	0.2295	0.1319	0.1319
6	0.4798	0.2512	0.1345	0.1345	21	0.4970	0.2390	0.1320	0.1320
7	0.4835	0.2627	0.1269	0.1269	25	0.4746	0.2594	0.1330	0.1330
10	0.4897	0.2501	0.1301	0.1301	29	0.5127	0.2331	0.1271	0.1271
12	0.5262	0.2240	0.1249	0.1249	36	0.5219	0.2273	0.1254	0.1254
14	0.5127	0.2363	0.1255	0.1255					

u(x_i) = 0.005 mole fr.; u(T) = 0.4 ºC [30 ºC; 70 ºC]; u(T) = 0.3 ºC [90 ºC]

Chemical kinetic data

Initial molar ratio AAc/HFBol=9/1 (laboratory stirred reactor)

Table S5: Dependence of the composition of the reaction mixture at different temperatures on the time of thermostating at atmospheric pressure, initial molar ratio AAc/HFBol = 9/1, $x_{H_2SO_4}$ = 0.01 mole fr. obtained in laboratory stirred reactor

**τ, min**	x_{AAc}	x_{HFBol}	x_{HFBAc}	x_{H_2O}	**τ, min**	x_{AAc}	x_{HFBol}	x_{HFBAc}	x_{H_2O}
				**T = 40 ºC, K_{eq}^{calc}= 0.0584**					
		*Set 1, $x_{H_2SO_4}$ = **0.0099** mole fr.*					*Set 2, $x_{H_2SO_4}$ = **0.0096** mole fr.*		
0	0.9005	0.0995	0	0	0	0.8997	0.1003	0	0
10	0.8982	0.0972	0.0023	0.0023	10	0.8968	0.0974	0.0029	0.0029
20	0.8953	0.0943	0.0052	0.0052	20	0.8943	0.0949	0.0054	0.0054
30	0.8930	0.0920	0.0075	0.0075	30	0.8926	0.0932	0.0071	0.0071
45	0.8908	0.0898	0.0097	0.0097	45	0.8893	0.0900	0.0104	0.0104
60	0.8886	0.0876	0.0119	0.0119	60	0.8871	0.0878	0.0125	0.0125
90	0.8836	0.0826	0.0169	0.0169	90	0.8821	0.0828	0.0175	0.0175
120	0.8808	0.0798	0.0197	0.0197	120	0.8801	0.0807	0.0196	0.0196
150	0.8756	0.0746	0.0249	0.0249	150	0.8679	0.0686	0.0317	0.0317
180	0.8733	0.0723	0.0272	0.0272	180	0.8719	0.0726	0.0278	0.0278
210	0.8715	0.0705	0.0290	0.0290	210	0.8741	0.0747	0.0256	0.0256
				**T = 50 ºC, K_{eq}^{calc}= 0.0669**					
		*Set 1, $x_{H_2SO_4}$ = **0.0108** mole fr.*					*Set 2, $x_{H_2SO_4}$ = **0.0099** mole fr.*		
0	0.9002	0.0998	0	0	0	0.9002	0.0998	0	0
10	0.8955	0.0951	0.0047	0.0047	10	0.8954	0.0950	0.0048	0.0048
20	0.8913	0.0909	0.0089	0.0089	20	0.8913	0.0909	0.0089	0.0089
30	0.8876	0.0872	0.0126	0.0126	30	0.8883	0.0879	0.0119	0.0119
45	0.8834	0.0830	0.0168	0.0168	45	0.8837	0.0833	0.0165	0.0165
60	0.8796	0.0792	0.0206	0.0206	60	0.8798	0.0794	0.0204	0.0204
90	0.8735	0.0731	0.0267	0.0267	90	0.8748	0.0744	0.0254	0.0254
120	0.8694	0.0690	0.0308	0.0308	120	0.8694	0.0690	0.0308	0.0308
150	0.8651	0.0647	0.0351	0.0351	150	0.8653	0.0649	0.0349	0.0349
180	0.8625	0.0621	0.0377	0.0377	180	0.8619	0.0615	0.0383	0.0383
210	0.8594	0.0590	0.0408	0.0408	210	0.8636	0.0632	0.0366	0.0366
				**T = 60 ºC, K_{eq}^{calc}= 0.0760**					
		*Set 1, $x_{H_2SO_4}$ = **0.0083** mole fr.*					*Set 2, $x_{H_2SO_4}$ = **0.0089** mole fr.*		
0	0.8997	0.1003	0	0	0	0.9000	0.1000	0	0
10	0.8920	0.0926	0.0077	0.0077	10	0.8926	0.0926	0.0074	0.0074

30	0.8810	0.0816	0.0187	0.0187	20	0.8866	0.0866	0.0134	0.0134
45	0.8759	0.0765	0.0238	0.0238	30	0.8817	0.0817	0.0183	0.0183
60	0.8708	0.0714	0.0289	0.0289	45	0.8765	0.0765	0.0235	0.0235
90	0.8618	0.0624	0.0379	0.0379	60	0.8708	0.0708	0.0292	0.0292
150	0.8540	0.0546	0.0457	0.0457	90	0.8644	0.0644	0.0356	0.0356
180	0.8553	0.0559	0.0444	0.0444	120	0.8585	0.0585	0.0415	0.0415
					150	0.8552	0.0552	0.0448	0.0448
210	0.8529	0.0535	0.0468	0.0468	180	0.8523	0.0523	0.0477	0.0477
					210	0.8514	0.0514	0.0486	0.0486
T = 70 °C, K_{eq}^{calc}= 0.0857									
Set 1, $x_{H_2SO_4} = \mathbf{0.0104}$ *mole fr.*					*Set 2,* $x_{H_2SO_4} = \mathbf{0.0104}$ *mole fr.*				
0	0.9000	0.1000	0	0	0	0.9016	0.0984	0	0
10	0.8852	0.0852	0.0148	0.0148	5	0.8940	0.0908	0.0076	0.0076
20	0.8754	0.0754	0.0246	0.0246	10	0.8866	0.0834	0.0150	0.0150
30	0.8689	0.0689	0.0311	0.0311	15	0.8810	0.0778	0.0206	0.0206
45	0.8596	0.0596	0.0404	0.0404	20	0.8763	0.0731	0.0253	0.0253
60	0.8553	0.0553	0.0447	0.0447	25	0.8727	0.0695	0.0289	0.0289
80	0.8498	0.0498	0.0502	0.0502	30	0.8686	0.0654	0.0330	0.0330
120	0.8461	0.0461	0.0539	0.0539	40	0.8633	0.0601	0.0383	0.0383
150	0.8460	0.0460	0.0540	0.0540	50	0.8604	0.0572	0.0412	0.0412
180	0.8437	0.0437	0.0563	0.0563	65	0.8577	0.0545	0.0439	0.0439
210	0.8423	0.0423	0.0577	0.0577	80	0.8535	0.0503	0.0481	0.0481
					100	0.8505	0.0473	0.0511	0.0511
240	0.8416	0.0416	0.0584	0.0584	130	0.8482	0.0450	0.0534	0.0534
					160	0.8476	0.0444	0.0540	0.0540
T = 80 °C, K_{eq}^{calc}= 0.0960									
Set 1, $x_{H_2SO_4} = \mathbf{0.0101}$ *mole fr.*					*Set 2,* $x_{H_2SO_4} = \mathbf{0.0107}$ *mole fr.*				
0	0.8997	0.1003	0	0	0	0.8992	0.1008	0	0
10	0.8775	0.0781	0.0222	0.0222	5	0.8881	0.0897	0.0111	0.0111
20	0.8659	0.0665	0.0338	0.0338	10	0.8789	0.0805	0.0203	0.0203
30	0.8565	0.0571	0.0432	0.0432	15	0.8707	0.0723	0.0285	0.0285
45	0.8491	0.0497	0.0506	0.0506	20	0.8676	0.0692	0.0316	0.0316
60	0.8455	0.0461	0.0542	0.0542	25	0.8615	0.0631	0.0377	0.0377
90	0.8410	0.0416	0.0587	0.0587	30	0.8574	0.0590	0.0418	0.0418
120	0.8402	0.0408	0.0595	0.0595	40	0.8521	0.0537	0.0471	0.0471
150	0.8404	0.0410	0.0593	0.0593	50	0.8491	0.0507	0.0501	0.0501
180	0.8402	0.0408	0.0595	0.0595	65	0.8458	0.0474	0.0534	0.0534
211	0.8399	0.0405	0.0598	0.0598	80	0.8446	0.0462	0.0546	0.0546
240	0.8430	0.0436	0.0567	0.0567	100	0.8428	0.0444	0.0564	0.0564
T = 90 °C, K_{eq}^{calc}= 0.1069									
Set 1, $x_{H_2SO_4} = \mathbf{0.0099}$ *mole fr.*					*Set 2,* $x_{H_2SO_4} = \mathbf{0.0106}$ *mole fr.*				
0	0.9000	0.1000	0	0	0	0.8994	0.1006	0	0
5	0.8815	0.0815	0.0185	0.0185	5	0.8786	0.0798	0.0208	0.0208
10	0.8719	0.0719	0.0281	0.0281	10	0.8679	0.0691	0.0315	0.0315
15	0.8632	0.0632	0.0368	0.0368	15	0.8596	0.0608	0.0398	0.0398
20	0.8570	0.0570	0.0430	0.0430	20	0.8535	0.0548	0.0459	0.0459
25	0.8542	0.0542	0.0458	0.0458	25	0.8497	0.0509	0.0497	0.0497
30	0.8519	0.0519	0.0481	0.0481	30	0.8462	0.0474	0.0532	0.0532
40	0.8476	0.0476	0.0524	0.0524	40	0.8436	0.0448	0.0558	0.0558
50	0.8448	0.0448	0.0552	0.0552	50	0.8429	0.0441	0.0565	0.0565
70	0.8432	0.0432	0.0568	0.0568	65	0.8406	0.0418	0.0588	0.0588
90	0.8441	0,0441	0.0559	0.0559	80	0.8402	0.0414	0.0592	0.0592
130	0.8437	0,0437	0.0563	0.0563	100	0.8383	0.0395	0.0611	0.0611

u(x_i) = 0.005 mole fr.; u(T) = 0.3 ºC

Initial molar ratio AAc/HFBol=9/1 (NMR apparatus)

Table S6: Dependence of the composition of the reaction mixture at different temperatures on the time of thermostating at atmospheric pressure, initial molar ratio AAc/HFBol = 9/1, $x_{H_2SO_4}$ = 0.01 mole fr. obtained in NMR apparatus

τ, min	x_{AAc}	x_{HFBol}	x_{HFBAc}	x_{H_2O}	τ, min	x_{AAc}	x_{HFBol}	x_{HFBAc}	x_{H_2O}
T = 40 °C; K_{eq}^{calc}= 0.0584									
Set 1; $x_{H_2SO_4} = \mathbf{0.0089}$ *mole fr.*					*Set 2;* $x_{H_2SO_4} = \mathbf{0.0096}$ *mole fr.*				
0	0.8927	0.0931	0.0071	0.0071	0	0.8943	0.0947	0.0055	0.0055

10	0.8918	0.0922	0.0080	0.0080	11	0.8930	0.0934	0.0068	0.0068
21	0.8904	0.0908	0.0094	0.0094	21	0.8915	0.0919	0.0083	0.0083
31	0.8893	0.0899	0.0104	0.0104	31	0.8902	0.0906	0.0096	0.0096
41	0.8885	0.0889	0.0113	0.0113	41	0.8890	0.0894	0.0108	0.0108
51	0.8875	0.0879	0.0123	0.0123	52	0.8875	0.0879	0.0123	0.0123
62	0.8854	0.0858	0.0144	0.0144	62	0.8869	0.0875	0.0128	0.0128
72	0.8855	0.0859	0.0143	0.0143	72	0.8856	0.0860	0.0142	0.0142
82	0.8834	0.0838	0.0164	0.0164	83	0.8844	0.0848	0.0154	0.0154
93	0.8825	0.0829	0.0173	0.0173	93	0.8834	0.0838	0.0164	0.0164
103	0.8810	0.0814	0.0188	0.0188	103	0.8817	0.0823	0.0180	0.0180
113	0.8802	0.0806	0.0196	0.0196	114	0.8816	0.0822	0.0181	0.0181
124	0.8800	0.0806	0.0197	0.0197	124	0.8807	0.0811	0.0191	0.0191
134	0.8785	0.0791	0.0212	0.0212	134	0.8797	0.0801	0.0201	0.0201
144	0.8770	0.0774	0.0228	0.0228	144	0.8790	0.0794	0.0208	0.0208
154	0.8769	0.0775	0.0228	0.0228	155	0.8785	0.0789	0.0213	0.0213
165	0.8764	0.0768	0.0234	0.0234	165	0.8781	0.0787	0.0216	0.0216
175	0.8748	0.0754	0.0249	0.0249	175	0.8773	0.0777	0.0225	0.0225
185	0.8738	0.0742	0.0260	0.0260	186	0.8750	0.0754	0.0248	0.0248
196	0.8738	0.0744	0.0259	0.0259	196	0.8742	0.0746	0.0256	0.0256
206	0.8734	0.0738	0.0264	0.0264	206	0.8742	0.0746	0.0256	0.0256
216	0.8729	0.0733	0.0269	0.0269	217	0.8731	0.0735	0.0267	0.0267
227	0.8729	0.0733	0.0269	0.0269	227	0.8727	0.0733	0.0270	0.0270
237	0.8714	0.0718	0.0284	0.0284	237	0.8722	0.0726	0.0276	0.0276
247	0.8717	0.0723	0.0280	0.0280	247	0.8716	0.0720	0.0282	0.0282
				**T = 45 °C; K_{eq}^{calc}= 0.0626**					
		Set 1; $x_{H_2SO_4} = \mathbf{0.0097}$ mole fr.					*Set 2; $x_{H_2SO_4} = \mathbf{0.0094}$ mole fr.*		
0	0.8920	0.0924	0.0078	0.0078	0	0.8930	0.0934	0.0068	0.0068
11	0.8894	0.0898	0.0104	0.0104	10	0.8908	0.0914	0.0089	0.0089
21	0.8871	0.0875	0.0127	0.0127	21	0.8894	0.0900	0.0103	0.0103
31	0.8855	0.0859	0.0143	0.0143	31	0.8869	0.0873	0.0129	0.0129
41	0.8837	0.0841	0.0161	0.0161	41	0.8855	0.0861	0.0142	0.0142
52	0.8805	0.0809	0.0193	0.0193	51	0.8838	0.0844	0.0159	0.0159
62	0.8790	0.0794	0.0208	0.0208	62	0.8826	0.0832	0.0171	0.0171
72	0.8764	0.0770	0.0233	0.0233	72	0.8813	0.0817	0.0185	0.0185
83	0.8762	0.0768	0.0235	0.0235	82	0.8799	0.0803	0.0199	0.0199
93	0.8745	0.0749	0.0253	0.0253	93	0.8786	0.0790	0.0212	0.0212
103	0.8734	0.0738	0.0264	0.0264	103	0.8773	0.0777	0.0225	0.0225
114	0.8722	0.0726	0.0276	0.0276	113	0.8773	0.0777	0.0225	0.0225
124	0.8703	0.0707	0.0295	0.0295	124	0.8758	0.0762	0.0240	0.0240
134	0.8696	0.0700	0.0302	0.0302	134	0.8751	0.0755	0.0247	0.0247
144	0.8676	0.0682	0.0321	0.0321	144	0.8747	0.0751	0.0251	0.0251
155	0.8679	0.0683	0.0319	0.0319	154	0.8718	0.0722	0.0280	0.0280
165	0.8664	0.0668	0.0334	0.0334	165	0.8712	0.0718	0.0285	0.0285
175	0.8658	0.0662	0.0340	0.0340	175	0.8709	0.0713	0.0289	0.0289
186	0.8636	0.0642	0.0361	0.0361	185	0.8694	0.0700	0.0303	0.0303
196	0.8637	0.0643	0.0360	0.0360	196	0.8680	0.0684	0.0318	0.0318
206	0.8633	0.0639	0.0364	0.0364	206	0.8680	0.0684	0.0318	0.0318
217	0.8629	0.0633	0.0369	0.0369	216	0.8676	0.0682	0.0321	0.0321
227	0.8622	0.0626	0.0376	0.0376	227	0.8666	0.0670	0.0332	0.0332
237	0.8606	0.0612	0.0391	0.0391	237	0.8660	0.0666	0.0337	0.0337
247	0.8605	0.0609	0.0393	0.0393	207	0.8651	0.0655	0.0347	0.0347
				Set 3; $x_{H_2SO_4} = \mathbf{0.0099}$ mole fr.					
0	0.8914	0.0918	0.0084	0.0084	134	0.8728	0.0732	0.0270	0.0270
10	0.8901	0.0905	0.0097	0.0097	144	0.8707	0.0711	0.0291	0.0291
21	0.8884	0.0888	0.0114	0.0114	154	0.8708	0.0714	0.0289	0.0289
31	0.8855	0.0861	0.0142	0.0142	165	0.8696	0.0700	0.0302	0.0302
41	0.8840	0.0844	0.0158	0.0158	175	0.8685	0.0691	0.0312	0.0312
51	0.8827	0.0833	0.0170	0.0170	185	0.8681	0.0685	0.0317	0.0317
62	0.8810	0.0814	0.0188	0.0188	195	0.8670	0.0674	0.0328	0.0328
72	0.8795	0.0801	0.0202	0.0202	206	0.8661	0.0667	0.0336	0.0336
82	0.8793	0.0797	0.0205	0.0205	216	0.8646	0.0650	0.0352	0.0352
93	0.8768	0.0774	0.0229	0.0229	227	0.8644	0.0648	0.0354	0.0354
103	0.8760	0.0766	0.0237	0.0237	237	0.8631	0.0635	0.0367	0.0367
113	0.8744	0.0748	0.0254	0.0254	247	0.8634	0.0640	0.0363	0.0363
124	0.8729	0.0735	0.0268	0.0268					
				**T = 50 °C; K_{eq}^{calc}= 0.0669**					
		Set 1; $x_{H_2SO_4} = \mathbf{0.0101}$ mole fr.					*Set 2; $x_{H_2SO_4} = \mathbf{0.0095}$ mole fr.*		

0	0.8909	0.0913	0.0089	0.0089	0	0.8917	0.0921	0.0081	0.0081
10	0.8887	0.0893	0.0110	0.0110	10	0.8895	0.0901	0.0102	0.0102
20	0.8860	0.0866	0.0137	0.0137	21	0.8882	0.0888	0.0115	0.0115
31	0.8846	0.0850	0.0152	0.0152	31	0.8869	0.0873	0.0129	0.0129
41	0.8821	0.0825	0.0177	0.0177	41	0.8843	0.0849	0.0154	0.0154
51	0.8811	0.0815	0.0187	0.0187	52	0.8822	0.0826	0.0176	0.0176
62	0.8794	0.0798	0.0204	0.0204	62	0.8805	0.0809	0.0193	0.0193
72	0.8773	0.0777	0.0225	0.0225	72	0.8785	0.0791	0.0212	0.0212
82	0.8760	0.0766	0.0237	0.0237	82	0.8775	0.0779	0.0223	0.0223
92	0.8751	0.0755	0.0247	0.0247	93	0.8762	0.0766	0.0236	0.0236
103	0.8732	0.0738	0.0265	0.0265	103	0.8757	0.0761	0.0241	0.0241
113	0.8729	0.0735	0.0268	0.0268	113	0.8748	0.0752	0.0250	0.0250
123	0.8717	0.0721	0.0281	0.0281	124	0.8746	0.0750	0.0252	0.0252
134	0.8706	0.0710	0.0292	0.0292	134	0.8722	0.0726	0.0276	0.0276
144	0.8698	0.0702	0.0300	0.0300	144	0.8719	0.0723	0.0279	0.0279
154	0.8688	0.0692	0.0310	0.0310	155	0.8712	0.0716	0.0286	0.0286
165	0.8674	0.0678	0.0324	0.0324	165	0.8707	0.0711	0.0291	0.0291
175	0.8636	0.0640	0.0362	0.0362	175	0.8660	0.0666	0.0337	0.0337
185	0.8664	0.0668	0.0334	0.0334	185	0.8690	0.0696	0.0307	0.0307
196	0.8654	0.0658	0.0344	0.0344	196	0.8680	0.0684	0.0318	0.0318
206	0.8619	0.0623	0.0379	0.0379	206	0.8656	0.0662	0.0341	0.0341
216	0.8609	0.0613	0.0389	0.0389	216	0.8661	0.0665	0.0337	0.0337
226	0.8609	0.0613	0.0389	0.0389	227	0.8658	0.0662	0.0340	0.0340
237	0.8600	0.0604	0.0398	0.0398	237	0.8612	0.0616	0.0386	0.0386
247	0.8620	0.0624	0.0378	0.0378	247	0.8645	0.0649	0.0353	0.0353
	Set 3; $x_{H_2SO_4} = \mathbf{0.0072}$ *mole fr.*					*Set 4;* $x_{H_2SO_4} = \mathbf{0.0097}$ *mole fr.*			
0	0.8963	0.0967	0.0035	0.0035	0	0.8900	0.0904	0.0098	0.0098
10	0.8928	0.0934	0.0069	0.0069	10	0.8861	0.0865	0.0137	0.0137
20	0.8898	0.0902	0.0100	0.0100	20	0.8836	0.0840	0.0162	0.0162
31	0.8874	0.0878	0.0124	0.0124	31	0.8817	0.0823	0.0180	0.0180
41	0.8848	0.0852	0.0150	0.0150	41	0.8804	0.0808	0.0194	0.0194
51	0.8824	0.0830	0.0173	0.0173	51	0.8780	0.0786	0.0217	0.0217
62	0.8800	0.0806	0.0197	0.0197	62	0.8765	0.0769	0.0233	0.0233
72	0.8778	0.0784	0.0219	0.0219	72	0.8759	0.0763	0.0239	0.0239
82	0.8753	0.0757	0.0245	0.0245	82	0.8724	0.0728	0.0274	0.0274
93	0.8738	0.0742	0.0260	0.0260	92	0.8694	0.0700	0.0303	0.0303
103	0.8711	0.0715	0.0287	0.0287	103	0.8690	0.0696	0.0307	0.0307
113	0.8705	0.0709	0.0293	0.0293	113	0.8692	0.0696	0.0306	0.0306
123	0.8692	0.0696	0.0306	0.0306	123	0.8687	0.0691	0.0311	0.0311
134	0.8664	0.0670	0.0333	0.0333	134	0.8678	0.0682	0.0320	0.0320
144	0.8655	0.0661	0.0342	0.0342	144	0.8643	0.0649	0.0354	0.0354
154	0.8645	0.0651	0.0352	0.0352	154	0.8648	0.0652	0.0350	0.0350
165	0.8642	0.0646	0.0356	0.0356	165	0.8645	0.0651	0.0352	0.0352
175	0.8626	0.0630	0.0372	0.0372	175	0.8618	0.0622	0.0380	0.0380
185	0.8623	0.0627	0.0375	0.0375	185	0.8617	0.0621	0.0381	0.0381
195	0.8622	0.0628	0.0375	0.0375	195	0.8619	0.0623	0.0379	0.0379
				**T = 55 °C; K_{eq}^{calc}= 0.0714**					
	Set 1; $x_{H_2SO_4} = \mathbf{0.0095}$ *mole fr.*					*Set 2;* $x_{H_2SO_4} = \mathbf{0.0100}$ *mole fr.*			
0	0.8920	0.0924	0.0078	0.0078	0	0.8904	0.0910	0.0093	0.0093
10	0.8885	0.0891	0.0112	0.0112	10	0.8876	0.0880	0.0122	0.0122
20	0.8860	0.0864	0.0138	0.0138	21	0.8847	0.0853	0.0150	0.0150
31	0.8829	0.0835	0.0168	0.0168	31	0.8804	0.0808	0.0194	0.0194
41	0.8792	0.0796	0.0206	0.0206	41	0.8803	0.0809	0.0194	0.0194
51	0.8779	0.0785	0.0218	0.0218	52	0.8778	0.0784	0.0219	0.0219
61	0.8772	0.0776	0.0226	0.0226	62	0.8752	0.0758	0.0245	0.0245
72	0.8750	0.0754	0.0248	0.0248	72	0.8732	0.0738	0.0265	0.0265
82	0.8735	0.0739	0.0263	0.0263	82	0.8717	0.0721	0.0281	0.0281
92	0.8719	0.0723	0.0279	0.0279	93	0.8695	0.0699	0.0303	0.0303
103	0.8701	0.0705	0.0297	0.0297	103	0.8678	0.0682	0.0320	0.0320
113	0.8685	0.0689	0.0313	0.0313	113	0.8671	0.0675	0.0327	0.0327
123	0.8638	0.0644	0.0359	0.0359	124	0.8658	0.0664	0.0339	0.0339
134	0.8645	0.0649	0.0353	0.0353	134	0.8653	0.0657	0.0345	0.0345
144	0.8634	0.0638	0.0364	0.0364	144	0.8643	0.0647	0.0355	0.0355
154	0.8622	0.0626	0.0376	0.0376	154	0.8619	0.0623	0.0379	0.0379
164	0.8611	0.0615	0.0387	0.0387	165	0.8616	0.0622	0.0381	0.0381
175	0.8600	0.0606	0.0397	0.0397	175	0.8589	0.0593	0.0409	0.0409
185	0.8592	0.0596	0.0406	0.0406	185	0.8570	0.0574	0.0428	0.0428

195	0.8583	0.0589	0.0414	0.0414	196	0.8587	0.0593	0.0410	0.0410
206	0.8604	0.0610	0.0393	0.0393	206	0.8556	0.0562	0.0441	0.0441
216	0.8605	0.0609	0.0393	0.0393	216	0.8587	0.0591	0.0411	0.0411
226	0.8584	0.0590	0.0413	0.0413	227	0.8559	0.0563	0.0439	0.0439
237	0.8593	0.0597	0.0405	0.0405	237	0.8553	0.0559	0.0444	0.0444
247	0.8586	0.0590	0.0412	0.0412	247	0.8545	0.0549	0.0453	0.0453
**T = 60 ºC; K_{eq}^{calc}= 0.0760**									
Set 1; $x_{H_2SO_4} = \mathbf{0.0088}$ *mole fr.*					*Set 2;* $x_{H_2SO_4} = \mathbf{0.0090}$ *mole fr.*				
0	0.8916	0.0920	0.0082	0.0082	0	0.8932	0.0936	0.0066	0.0066
10	0.8886	0.0892	0.0111	0.0111	10	0.8899	0.0903	0.0099	0.0099
20	0.8851	0.0855	0.0147	0.0147	20	0.8857	0.0861	0.0141	0.0141
31	0.8825	0.0829	0.0173	0.0173	31	0.8835	0.0839	0.0163	0.0163
41	0.8796	0.0800	0.0202	0.0202	41	0.8798	0.0804	0.0199	0.0199
51	0.8776	0.0780	0.0222	0.0222	51	0.8787	0.0793	0.0210	0.0210
61	0.8753	0.0757	0.0245	0.0245	61	0.8758	0.0762	0.0240	0.0240
72	0.8743	0.0747	0.0255	0.0255	72	0.8748	0.0752	0.0250	0.0250
82	0.8721	0.0725	0.0277	0.0277	82	0.8711	0.0717	0.0286	0.0286
92	0.8696	0.0700	0.0302	0.0302	92	0.8712	0.0716	0.0286	0.0286
103	0.8689	0.0693	0.0309	0.0309	103	0.8707	0.0711	0.0291	0.0291
113	0.8659	0.0663	0.0339	0.0339	113	0.8670	0.0674	0.0328	0.0328
123	0.8673	0.0677	0.0325	0.0325	123	0.8660	0.0666	0.0337	0.0337
133	0.8665	0.0669	0.0333	0.0333	134	0.8648	0.0654	0.0349	0.0349
144	0.8646	0.0650	0.0352	0.0352	144	0.8652	0.0658	0.0345	0.0345
154	0.8624	0.0628	0.0374	0.0374	154	0.8636	0.0640	0.0362	0.0362
164	0.8613	0.0617	0.0385	0.0385	164	0.8610	0.0614	0.0388	0.0388
175	0.8618	0.0622	0.0380	0.0380	175	0.8621	0.0625	0.0377	0.0377
185	0.8594	0.0600	0.0403	0.0403	185	0.8602	0.0608	0.0395	0.0395
195	0.8583	0.0587	0.0415	0.0415	195	0.8591	0.0595	0.0407	0.0407
206	0.8580	0.0584	0.0418	0.0418	206	0.8572	0.0578	0.0425	0.0425
216	0.8583	0.0587	0.0415	0.0415	216	0.8577	0.0583	0.0420	0.0420
226	0.8566	0.0570	0.0432	0.0432	226	0.8567	0.0573	0.0430	0.0430
237	0.8561	0.0565	0.0437	0.0437	237	0.8563	0.0567	0.0435	0.0435
247	0.8554	0.0558	0.0444	0.0444	247	0.8559	0.0563	0.0439	0.0439
**T = 65 ºC; K_{eq}^{calc}= 0.0808**									
Set 1; $x_{H_2SO_4} = \mathbf{0.0087}$ *mole fr.*					*Set 2;* $x_{H_2SO_4} = \mathbf{0.0103}$ *mole fr.*				
0	0.8908	0.0912	0.0090	0.0090	0	0.8890	0.0896	0.0107	0.0107
10	0.8861	0.0865	0.0137	0.0137	10	0.8855	0.0859	0.0143	0.0143
21	0.8824	0.0828	0.0174	0.0174	21	0.8821	0.0825	0.0177	0.0177
31	0.8795	0.0799	0.0203	0.0203	31	0.8792	0.0796	0.0206	0.0206
41	0.8764	0.0768	0.0234	0.0234	41	0.8761	0.0765	0.0237	0.0237
52	0.8737	0.0743	0.0260	0.0260	51	0.8727	0.0731	0.0271	0.0271
62	0.8717	0.0721	0.0281	0.0281	61	0.8708	0.0712	0.0290	0.0290
72	0.8700	0.0706	0.0297	0.0297	72	0.8669	0.0675	0.0328	0.0328
82	0.8673	0.0677	0.0325	0.0325	82	0.8664	0.0668	0.0334	0.0334
93	0.8661	0.0667	0.0336	0.0336	92	0.8659	0.0663	0.0339	0.0339
103	0.8641	0.0645	0.0357	0.0357	103	0.8633	0.0637	0.0365	0.0365
113	0.8618	0.0624	0.0379	0.0379	113	0.8616	0.0620	0.0382	0.0382
124	0.8604	0.0608	0.0394	0.0394	124	0.8602	0.0606	0.0396	0.0396
134	0.8598	0.0602	0.0400	0.0400	133	0.8601	0.0605	0.0397	0.0397
144	0.8579	0.0583	0.0419	0.0419	144	0.8589	0.0593	0.0409	0.0409
155	0.8576	0.0580	0.0422	0.0422	154	0.8579	0.0585	0.0418	0.0418
165	0.8564	0.0570	0.0433	0.0433	164	0.8563	0.0567	0.0435	0.0435
175	0.8558	0.0562	0.0440	0.0440	175	0.8557	0.0561	0.0441	0.0441
186	0.8540	0.0544	0.0458	0.0458	185	0.8542	0.0548	0.0455	0.0455
196	0.8540	0.0546	0.0457	0.0457	195	0.8530	0.0536	0.0467	0.0467
206	0.8534	0.0538	0.0464	0.0464	206	0.8543	0.0547	0.0455	0.0455
217	0.8526	0.0530	0.0472	0.0472	216	0.8509	0.0513	0.0489	0.0489
227	0.8521	0.0525	0.0477	0.0477	226	0.8501	0.0505	0.0497	0.0497
237	0.8516	0.0522	0.0481	0.0481	237	0.8502	0.0508	0.0495	0.0495
247	0.8503	0.0507	0.0495	0.0495	247	0.8502	0.0506	0.0496	0.0496
**T = 70 ºC; K_{eq}^{calc}= 0.0857**									
Set 1; $x_{H_2SO_4} = \mathbf{0.0080}$ *mole fr.*					*Set 2;* $x_{H_2SO_4} = \mathbf{0.0089}$ *mole fr.*				
0	0.8908	0.0912	0.0090	0.0090	0	0.8855	0.0859	0.0143	0.0143
10	0.8853	0.0857	0.0145	0.0145	10	0.8803	0.0809	0.0194	0.0194
21	0.8814	0.0818	0.0184	0.0184	21	0.8763	0.0767	0.0235	0.0235
31	0.8769	0.0775	0.0228	0.0228	31	0.8732	0.0736	0.0266	0.0266
41	0.8743	0.0747	0.0255	0.0255	41	0.8703	0.0707	0.0295	0.0295

51	0.8715	0.0721	0.0282	0.0282	51	0.8675	0.0679	0.0323	0.0323
62	0.8679	0.0683	0.0319	0.0319	62	0.8659	0.0663	0.0339	0.0339
72	0.8655	0.0659	0.0343	0.0343	72	0.8629	0.0633	0.0369	0.0369
82	0.8637	0.0643	0.0360	0.0360	82	0.8613	0.0617	0.0385	0.0385
92	0.8624	0.0628	0.0374	0.0374	93	0.8599	0.0603	0.0399	0.0399
103	0.8613	0.0617	0.0385	0.0385	103	0.8582	0.0586	0.0416	0.0416
113	0.8592	0.0596	0.0406	0.0406	113	0.8554	0.0558	0.0444	0.0444
123	0.8581	0.0585	0.0417	0.0417	123	0.8558	0.0562	0.0440	0.0440
134	0.8549	0.0553	0.0449	0.0449	134	0.8549	0.0553	0.0449	0.0449
144	0.8544	0.0548	0.0454	0.0454	144	0.8513	0.0519	0.0484	0.0484
154	0.8546	0.0550	0.0452	0.0452	154	0.8535	0.0539	0.0463	0.0463
165	0.8537	0.0541	0.0461	0.0461	165	0.8497	0.0503	0.0500	0.0500
175	0.8522	0.0526	0.0476	0.0476	175	0.8498	0.0502	0.0500	0.0500
185	0.8502	0.0506	0.0496	0.0496	185	0.8514	0.0518	0.0484	0.0484
196	0.8502	0.0506	0.0496	0.0496	196	0.8487	0.0493	0.0510	0.0510
206	0.8494	0.0500	0.0503	0.0503	206	0.8480	0.0486	0.0517	0.0517
216	0.8509	0.0513	0.0489	0.0489	216	0.8486	0.0492	0.0511	0.0511
226	0.8495	0.0501	0.0502	0.0502	227	0.8496	0.0500	0.0502	0.0502
237	0.8493	0.0499	0.0504	0.0504	237	0.8465	0.0469	0.0533	0.0533
247	0.8459	0.0465	0.0538	0.0538	247	0.8467	0.0473	0.0530	0.0530

u(x_i) = 0.005 mole fr.; u(T) = 0.4 ºC

Initial molar ratio AAc/HFBol=65/35 (laboratory stirred reactor)

Table S7: Dependence of the composition of the reaction mixture at different temperatures on the time of thermostating at atmospheric pressure, initial molar ratio AAc/HFBol = 65/35, $x_{H_2SO_4}$ = 0.01 mole fr. obtained in laboratory stirred reactor

τ, min	x_{AAc}	x_{HFBol}	x_{HFBAc}	x_{H_2O}	τ, min	x_{AAc}	x_{HFBol}	x_{HFBAc}	x_{H_2O}
T = 30 ºC; K_{eq}^{calc}= 0.0755									
Set 1; $x_{H_2SO_4}$ = 0.0148 mole fr.					Set 2; $x_{H_2SO_4}$ = 0.0111 mole fr.				
0	0.6499	0.3501	0	0	0	0.6497	0.3503	0	0
10	0.6438	0.3438	0.0062	0.0062	10	0.6466	0.3472	0.0031	0.0031
20	0.6402	0.3402	0.0098	0.0098	20	0.6439	0.3443	0.0059	0.0059
30	0.6347	0.3349	0.0152	0.0152	30	0.6406	0.3410	0.0092	0.0092
45	0.6303	0.3303	0.0197	0.0197	45	0.6394	0.3398	0.0104	0.0104
60	0.6267	0.3267	0.0233	0.0233	60	0.6349	0.3353	0.0149	0.0149
90	0.6164	0.3164	0.0336	0.0336	90	0.6311	0.3317	0.0186	0.0186
120	0.6096	0.3096	0.0404	0.0404	120	0.6260	0.3266	0.0237	0.0237
150	0.6027	0.3027	0.0473	0.0473	150	0.6210	0.3214	0.0288	0.0288
180	0.6021	0.3021	0.0479	0.0479	180	0.6175	0.3181	0.0322	0.0322
210	0.5973	0.2975	0.0526	0.0526	210	0.6133	0.3137	0.0365	0.0365
T = 50 ºC; K_{eq}^{calc}= 0.0942									
Set 1; $x_{H_2SO_4}$ = 0.0101 mole fr.					Set 2; $x_{H_2SO_4}$ = 0.0097 mole fr.				
0	0.6499	0.3501	0	0	0	0.6505	0.3495	0	0
10	0.6364	0.3366	0.0135	0.0135	10	0.6393	0.3383	0.0112	0.0112
20	0.6279	0.3281	0.0220	0.0220	20	0.6329	0.3321	0.0175	0.0175
30	0.6192	0.3194	0.0307	0.0307	30	0.6245	0.3235	0.0260	0.0260
45	0.6074	0.3076	0.0425	0.0425	45	0.6158	0.3148	0.0347	0.0347
60	0.5899	0.2901	0.0600	0.0600	60	0.6081	0.3073	0.0423	0.0423
90	0.5933	0.2935	0.0566	0.0566	90	0.5960	0.2952	0.0544	0.0544
120	0.5805	0.2807	0.0694	0.0694	120	0.5884	0.2874	0.0621	0.0621
150	0.5719	0.2721	0.0780	0.0780	150	0.5809	0.2801	0.0695	0.0695
180	0.5636	0.2638	0.0863	0.0863	180	0.5783	0.2773	0.0722	0.0722
210	0.5644	0.2646	0.0855	0.0855	210	0.5713	0.2703	0.0792	0.0792
T = 70 ºC; K_{eq}^{calc}= 0.1144									
Set 1; $x_{H_2SO_4}$ = 0.0102 mole fr.					Set 2; $x_{H_2SO_4}$ = 0.0113 mole fr.				
0	0.6501	0.3499	0	0	0	0.6505	0.3495	0	0
10	0.6501	0.3499	0.0000	0.0000	10.5	0.6114	0.3104	0.0391	0.0391
20	0.5434	0.2432	0.1067	0.1067	20	0.5945	0.2935	0.0560	0.0560
30	0.5642	0.2640	0.0859	0.0859	30	0.5791	0.2781	0.0714	0.0714
45	0.5684	0.2682	0.0817	0.0817	45.5	0.5658	0.2648	0.0847	0.0847
60	0.4973	0.1971	0.1528	0.1528	60	0.5579	0.2569	0.0926	0.0926
91	0.5423	0.2421	0.1078	0.1078	91	0.5399	0.2389	0.1106	0.1106
120	0.5363	0.2361	0.1138	0.1138	120	0.5411	0.2401	0.1094	0.1094

150	0.5624	0.2622	0.0877	0.0877	150	0.5323	0.2313	0.1182	0.1182
180	0.4802	0.1800	0.1699	0.1699	180	0.5358	0.2348	0.1147	0.1147
					210	0.5333	0.2323	0.1172	0.1172
**T = 90 ºC; K_{eq}^{calc}= 0.1361**									
Set 1; $x_{H_2SO_4} = 0.0110$ mole fr.					Set 2; $x_{H_2SO_4} = 0.0116$ mole fr.				
0	0.6499	0.3501	0	0	0	0.6487	0.3513	0	0
10.5	0.5735	0.2737	0.0764	0.0764	10	0.5799	0.2789	0.0706	0.0706
20	0.5433	0.2435	0.1066	0.1066	20	0.5535	0.2535	0.0965	0.0965
30	0.5429	0.2431	0.1070	0.1070	30	0.5418	0.2418	0.1082	0.1082
45.5	0.5267	0.2269	0.1232	0.1232	45	0.5348	0.2332	0.1160	0.1160
60	0.5270	0.2272	0.1229	0.1229	60	0.5285	0.2281	0.1217	0.1217
91	0.5183	0.2185	0.1316	0.1316	90	0.5301	0.2301	0.1199	0.1199
120	0.5287	0.2289	0.1212	0.1212	120	0.5272	0.2278	0.1225	0.1225
150	0.5314	0.2316	0.1185	0.1185	150	0.5274	0.2280	0.1223	0.1223
180	0.5178	0.2180	0.1321	0.1321	180	0.5271	0.2275	0.1227	0.1227
210	0.5324	0.2326	0.1175	0.1175	210	0.5263	0.2279	0.1229	0.1229

u(x_i) = 0.005 mole fr.; u(T) = 0.3 ºC

Combined standard uncertainties

Table S8: The procedure for calculating the combined standard uncertainties.

Overall equation view $y = a \cdot x + b$	
$x = \frac{1}{T}$	$u(x) = \frac{\sigma(T_i)}{{T_i}^2}$
$x_i - x_{av}$	$u(x_i - x_{av}) = \sqrt{u^2(x_i) + \frac{\sqrt{\sum u^2(x_i)}}{n}}$
$(x_i - x_{av})^2$	$u((x_i - x_{av})^2) = (x_i - x_{av}) \cdot u(x_i - x_{av}) \cdot \sqrt{2}$
	$u(y_i) = \frac{\sum \lvert k_i - k_{av} \rvert}{n \cdot k_i}$
$y = lnk$	
$y_i - y_{av}$	$u(y_i - y_{av}) = \sqrt{u^2(y_i) + \frac{\sqrt{\sum u^2(y_i)}}{n}}$
$(y_i - y_{av})^2$	$u((y_i - y_{av})^2) = (y_i - y_{av}) \cdot u(y_i - y_{av}) \cdot \sqrt{2}$
$(x_i - x_{av}) \cdot (y_i - y_{av})$	$u((x_i - x_{av}) \cdot (y_i - y_{av})) = (x_i - x_{av}) \cdot (y_i - y_{av}) \cdot \sqrt{\left(\frac{u(x_i - x_{av})}{x_i - x_{av}}\right)^2 + \left(\frac{u(y_i - y_{av})}{y_i - y_{av}}\right)^2}$
$r = \frac{\sum((x_i - x_{av}) \cdot (y_i - y_{av}))}{\sqrt{\sum(x_i - x_{av})^2 \cdot \sum(y_i - y_{av})^2}}$	$u(r) = r \cdot \sqrt{\frac{\sum u^2((x_i - x_{av}) \cdot (y_i - y_{av}))}{(\sum(x_i - x_{av}) \cdot (y_i - y_{av}))^2} + \left(\frac{\sum u^2(y_i - y_{av})^2}{2 \cdot \sum(y_i - y_{av})^2}\right)^2 + \left(\frac{\sum u^2(x_i - x_{av})^2}{2 \cdot \sum(x_i - x_{av})^2}\right)^2}$
$a = r \cdot \sqrt{\frac{\sum(y_i - y_{av})^2}{\sum(x_i - x_{av})^2}}$	$u(a) = a \cdot \sqrt{\left(\frac{u(r)}{r}\right)^2 + \left(\frac{\sum u^2(y_i - y_{av})^2}{2 \cdot \sum(y_i - y_{av})^2}\right)^2 + \left(\frac{\sum u^2(x_i - x_{av})^2}{2 \cdot \sum(x_i - x_{av})^2}\right)^2}$
$a \cdot x_{av}$	$u(a \cdot x_{av}) = a \cdot x_{av} \cdot \sqrt{\left(\frac{u(a)}{a}\right)^2 + \frac{\sum u^2(x_i)}{(\sum(x_i))^2}}$
$b = y_{av} - a \cdot x_{av}$	$u(b) = \sqrt{u^2(a \cdot x_{av}) + \frac{\sum u^2 y_i}{n^2}}$

Where T – temperature; u – uncertainty distribution; n – number of experiments; k – reaction rate constant; $\sigma(T) = 0.3$ °C; r – determination coefficient; $a = \frac{-E_A}{R}$, where E_A – activation energy, R – gas constant; $b = lnA$, where A – pre-exponential factor.
